# SMG6 localizes to the chromatoid body and shapes the male germ cell transcriptome to drive spermatogenesis

**DOI:** 10.1093/nar/gkac900

**Published:** 2022-10-19

**Authors:** Tiina Lehtiniemi, Matthieu Bourgery, Lin Ma, Ammar Ahmedani, Margareeta Mäkelä, Juho Asteljoki, Opeyemi Olotu, Samuli Laasanen, Fu-Ping Zhang, Kun Tan, Jennifer N Chousal, Dana Burow, Satu Koskinen, Asta Laiho, Laura L Elo, Frédéric Chalmel, Miles F Wilkinson, Noora Kotaja

**Affiliations:** Institute of Biomedicine, Integrative Physiology and Pharmacology Unit, University of Turku, Turku, Finland; Institute of Biomedicine, Integrative Physiology and Pharmacology Unit, University of Turku, Turku, Finland; Institute of Biomedicine, Integrative Physiology and Pharmacology Unit, University of Turku, Turku, Finland; Institute of Biomedicine, Integrative Physiology and Pharmacology Unit, University of Turku, Turku, Finland; Institute of Biomedicine, Integrative Physiology and Pharmacology Unit, University of Turku, Turku, Finland; Institute of Biomedicine, Integrative Physiology and Pharmacology Unit, University of Turku, Turku, Finland; Institute of Biomedicine, Integrative Physiology and Pharmacology Unit, University of Turku, Turku, Finland; Institute of Biomedicine, Integrative Physiology and Pharmacology Unit, University of Turku, Turku, Finland; Institute of Biomedicine, Integrative Physiology and Pharmacology Unit, University of Turku, Turku, Finland; Turku Center for Disease Modeling, University of Turku, Turku, Finland; GM-Unit, Helsinki Institute of Life Science, Faculty of Medicine, University of Helsinki, Helsinki, Finland; Department of Obstetrics, Gynecology, and Reproductive Sciences, School of Medicine, University of California San Diego, La Jolla, CA 92093, USA; Department of Obstetrics, Gynecology, and Reproductive Sciences, School of Medicine, University of California San Diego, La Jolla, CA 92093, USA; Department of Obstetrics, Gynecology, and Reproductive Sciences, School of Medicine, University of California San Diego, La Jolla, CA 92093, USA; Turku Bioscience Centre, University of Turku and Åbo Akademi University, Turku, Finland; Turku Bioscience Centre, University of Turku and Åbo Akademi University, Turku, Finland; Institute of Biomedicine, Integrative Physiology and Pharmacology Unit, University of Turku, Turku, Finland; Turku Bioscience Centre, University of Turku and Åbo Akademi University, Turku, Finland; University of Rennes, Inserm, EHESP, Irset (Institut de recherche en santé, environnement et travail) - UMR_S 1085, F-35000, Rennes, France; Department of Obstetrics, Gynecology, and Reproductive Sciences, School of Medicine, University of California San Diego, La Jolla, CA 92093, USA; Institute for Genomic Medicine (IGM), University of California, San Diego, La Jolla, CA 92093, USA; Institute of Biomedicine, Integrative Physiology and Pharmacology Unit, University of Turku, Turku, Finland

## Abstract

Nonsense-mediated RNA decay (NMD) is a highly conserved and selective RNA turnover pathway that depends on the endonuclease SMG6. Here, we show that SMG6 is essential for male germ cell differentiation in mice. Germ-cell conditional knockout (cKO) of *Smg6* induces extensive transcriptome misregulation, including a failure to eliminate meiotically expressed transcripts in early haploid cells, and accumulation of NMD target mRNAs with long 3′ untranslated regions (UTRs). Loss of SMG6 in the male germline results in complete arrest of spermatogenesis at the early haploid cell stage. We find that SMG6 is strikingly enriched in the chromatoid body (CB), a specialized cytoplasmic granule in male germ cells also harboring PIWI-interacting RNAs (piRNAs) and the piRNA-binding protein PIWIL1. This raises the possibility that SMG6 and the piRNA pathway function together, which is supported by several findings, including that *Piwil1*-KO mice phenocopy *Smg6*-cKO mice and that SMG6 and PIWIL1 co-regulate many genes in round spermatids. Together, our results demonstrate that SMG6 is an essential regulator of the male germline transcriptome, and highlight the CB as a molecular platform coordinating RNA regulatory pathways to control sperm production and fertility.

## INTRODUCTION

Spermatogenesis is a tightly regulated process that ensures the continuous production of spermatozoa from spermatogonial stem cells (1,2). Each step of spermatogenesis is governed by temporally regulated gene expression programs resulting in major cell type-specific shifts in transcriptome composition (3–5). Differentiating male germ cells, particularly meiotic spermatocytes and postmeiotic haploid round spermatids, have exceptionally diverse transcriptomes (6–8). The transcriptomes of these cells not only consist of gene products supporting the unique processes taking place during and after meiosis (9), but also an unusually diverse set of unannotated transcripts from intergenic genomic regions (6–8). This widespread genome transcription has been shown to serve important functions for example in modulating mutation rates in a gene-specific manner on the basis of germline expression through transcription-coupled DNA repair (10). Pervasive transcription creates a high demand for posttranscriptional regulatory mechanisms to monitor the quality of transcriptome and eliminate unnecessary and aberrant transcripts. In parallel, translational regulation becomes prominent when transcripts produced in meiotic and postmeiotic cells need to be translationally repressed and stored for later use in condensing spermatids that are transcriptionally inactive due to the replacement of histones with protamines (11,12).

Coinciding with active posttranscriptional regulation, unusually large cytoplasmic ribonucleoprotein (RNP) granules, called germ granules, appear in male germ cells (13,14). Although the exact functions of germ granules have remained enigmatic, they are known to accumulate diverse RNA species, scaffold proteins, and RNA regulatory proteins, which supports their role as platforms for posttranscriptional regulatory processes (13). Histologically, the most prominent germ granules in developing male germ cells are the intermitochondrial cement (IMC) and the chromatoid body (CB), both of which first appear in spermatocytes at a time point when a remarkably large percentage of the genome is transcribed (13). The IMC disintegrates before meiotic divisions occur in spermatocytes, while the CB persists, condensing to its final form in early round spermatids (13).

Molecular characterization of the IMC and the CB has revealed they exhibit a strong link with the PIWI-interacting (piRNA) pathway, a pathway driven by the largest known class of small non-coding RNAs expressed in animal cells (13,15,16). piRNAs are 24–35 nucleotides in length and associate with the PIWI subfamily of Argonaute proteins (17,18). Distinct types of piRNAs are expressed in the mouse germline. Fetal piRNAs associate with PIWIL2 (MILI) and PIWIL4 (MIWI2), and have well-established function in transposon silencing (17,19). In postnatal germ cells, piRNAs can be broadly divided into two classes: ‘pre-pachytene’ and ‘pachytene’. Pre-pachytene piRNAs are expressed already in early spermatogenic cells before the pachytene phase of the first meiotic division, and they mostly derive from genomic clusters overlapping with protein-coding genes (17,20). Pachytene piRNA expression is induced in pachytene spermatocytes. They derive from large intergenic clusters and they mainly associate with PIWIL1 (MIWI) (17,21). Pre-pachytene and pachytene piRNAs are largerly uncharacterized functionally; some are involved in translational control and destabilization of mRNAs and long non-coding RNAs (22–27). The protein composition of germ granules suggest that pachytene piRNA biogenesis primarily occurs in the IMC, followed by a transfer of PIWIL1-loaded piRNAs to the CB for their downstream functions (13). Consistent with the CB serving as a site of action for the piRNA pathway, the CB is highly enriched in piRNAs, PIWI proteins, and a diverse set of long non-coding RNAs and mRNAs that are potential targets for piRNAs (15).

In addition to piRNA pathway components, the CB accumulates several proteins critical for the nonsense-mediated RNA decay (NMD) pathway (15). This translation-dependent RNA turnover mechanism destabilizes target RNAs that harbor a main open-reading frame (ORF) terminated by a stop codon associated with a decay-inducing signal (28). Most NMD target RNAs can be divided into two classes: (i) aberrant RNAs harboring premature termination codons that encode truncated proteins and (ii) normal mRNAs encoding full-length proteins that are defined by a stop codon in specific contexts that elicit NMD (29,30). Therefore, NMD is considered both a quality control pathway and a regulator of normal gene expression dynamics (28,31,32). A large constellation of proteins critical for NMD has been identified, including the up-frameshift (UPF) core machinery (UPF1, UPF2 and UPF3B) and the exon junction complex (EJC). After an RNA is recognized as being a NMD target, a step involving phosphorylation of UFP1 by the kinase Suppressor with Morphogenetic effect on Genitalia 1 (SMG1), the decay of RNA is mediated by distinct degradation mechanisms. One of these mechanisms includes the SMG5-SMG7 heterodimer, which is recruited to RNAs harboring phosphorylated UPF1 and promotes RNA deadenylation, followed by exonucleolytic decay (33). The other degradation mechanism depends on the endonuclease, SMG6, which interacts with UPF1 and catalyzes a single-stranded cleavage of the RNA in the vicinity of the NMD-triggering stop codon (33).

We have shown that the CB accumulates several NMD factors, including SMG6, UPF1, SMG1 and several core EJC proteins (15). SMG6 has a particularly prominent CB localization (15), and thus we chose to examine its functional role in the male germline. Given that SMG6 is required for embryonic development (34), we generated a germ cell-specific conditional *Smg6* knockout (KO) mouse line to dissect the function of SMG6 in male germ cells. Analysis of these *Smg6* conditional (c) KO mice revealed that SMG6 is essential for male fertility and the differentiation of round spermatids - the germ cell stage that harbors mature CBs. We found that SMG6 functions in downregulating several classes of mRNAs in germ cells, including NMD target mRNAs harboring long 3′UTRs and mRNAs expressed during the previous stage of spermatogenesis: meiosis. We also showed that SMG6 share target RNAs with the piRNA-binding protein PIWIL1. This finding, coupled with the strikingly similar spermatogenic defects in *Smg6*- and *Piwil1*-mutant mice, led us to determine the interconnection of the NMD and piRNA pathways in the CB. Our results suggest that in germ cells, the CB participates in the function of NMD and piRNA pathway to regulate long 3′UTR-containing transcripts and support the development of haploid germ cell to form fertile spermatozoa.

## MATERIALS AND METHODS

### Animals

Mice were maintained and housed at the central animal facility of the University of Turku, Finland, under controlled pathogen-free conditions, following local laws and regulations (Finnish Act on the Protection of Animals Used for Scientific or Educational Purposes [497/2013], Government Decree on the Protection of Animals Used for Scientific or Educational Purposes [564/2013]). Mice were euthanized by CO_2_ inhalation followed by cervical dislocation. The Laboratory Animal Care and Use Committee of the University of Turku approved all the animal experiments.

### Protein extraction from mouse tissues

Tissue samples were homogenized in RIPA lysis buffer (50 mM Tris–HCl at pH 7.5, 1% Triton X-100, 0.5% w/v sodium deoxycholate, 0.05% w/v sodium dodecyl sulfate, 1 mM EDTA, 150 mM NaCl) supplemented with 1 mM DTT, 0.2 mM PMSF and 1× protease inhibitor cocktail, and the lysates were cleared by centrifugation at 14 000 × g for 10 min. For tissue expression and ontogenesis studies, protein concentration was measured using Pierce BCA protein assay kit (Life Technologies, 23227); absorbance was measured with a Victor2 plate reader (Wallac, Turku, Finland). Samples diluted in Laemmli buffer were incubated 5 min at 95°C before western blotting.

### Western blotting

Proteins were separated by 10% SDS-PAGE and transferred to PVDF membranes (Amersham, RPN303F) with wet-blotting system (Bio-Rad). After blocking in 100% methanol, membranes were air-dried overnight at room temperature, and then incubated in primary antibodies diluted in 5% skimmed milk, 0.1% triton X-100, 1× TBS overnight at +4°C. Horseradish Peroxide (HRP)-conjugated anti-rabbit or anti-mouse IgG was used as a secondary antibody (dilution of 1:1000). Proteins were detected using western lightening ECL pro (Ne112200IEA, Perkin Elmer, Netherlands). Signals were obtained with LAS4000 (FujiFilm), saved as 16-bit TIFF files and processed with ImageJ software version 1.8.0 (National Institute of Health, USA) and Adobe Photoshop.

### Immunohistochemistry

Testis from juvenile wt (C57BL/6) or adult *Smg6-*cKO and control mice were collected and fixed in 4% phosphate buffered formaldehyde overnight, dehydrated, embedded in paraffin and cut into sections. Testis sections were rehydrated with 3 × 5 min in xylene, 2 × 10 min in 100% ethanol (EtOH), 2 × 10 min in 96% EtOH, 2 × 10 min in 70% EtOH and 5 min in ddH_2_O and antigens were retrieved by pressure cooking in 10 mM sodium citrate (pH 6.0) for 2 h. Non-specific binding was blocked with 30 min incubation in 3% BSA in 0.5% PBST (blocking solution). Primary antibodies were diluted in blocking solution (1:200 form anti-EST1A/SMG6, Abcam ab87539) and sections were incubated at +4°C overnight. Slides were washed 2 × 5 min with 0.05% PBST and endogenous peroxidase activity was blocked with 20 min incubation in 3% H_2_O_2_. Secondary antibody incubation was done using Envision + system with HRP labeled polymer anti-rabbit (Dako) for 30 min at room temperature. DAB-color formation was detected with 3,3′-diaminobenzidine (liquid DAB+, Dako) and color reaction was stop with 3 × 3 min dH_2_O wash. Sections were stained with 10 s incubation in Mayer's Hematoxylin and washed under running water for 5 min. Sections were dehydrated with 2 × 5 min in 70% EtOH, 2 × 10 min in 96% EtOH, 2 × 5 min in 100% EtOH and 3 × 5 min in xylene and mounted with PERTEX medium. Images were taken with digital slide scanner Panoramic 250 Flash III (3DHistech) and processed using Adobe Photoshop.

### Immunofluorescence staining

Samples of human testis and intact adult *Smg6-*cKO, *Piwil1-*KO and control mouse testes were fixed in 4% phosphate buffered formaldehyde overnight. The fixed samples were dehydrated, embedded into paraffin and cut to sections. Paraffin-embedded testis sections were deparaffinized by incubating 3 × 5 min in xylene, 2 × 10 min in 100% ethanol, 2 × 10 min in 96% ethanol, 2 × 10 min in 70% ethanol and then washed in milliQ water 2 × 2 min. Sections were subjected to antigen retrieval in sodium citrate solution (10 mM sodium citrate, 0.05% Tween 20 [Sigma, P2287], pH 6.0) or in Tris–EDTA solution (10 mM Tris base, 1 mM EDTA Solution, 0.05% Tween 20, pH 9.0) by boiling in pressure cooker (at 120°C, 20 min). Slides were incubated at 10% normal donkey serum (Jackson Immunoresearch, 017-000-121) and 3% bovine serum albumin (Sigma, A2153) in PBST (0.1% Tween-20) (= blocking solution) for 1 h. Primary antibodies were diluted in blocking solution (1:100–1:500) and incubation was performed overnight at 4°C in humidified environment. Acrosome labeling was done with Rhodamine-conjugated peanut agglutinin (PNA, RL-1072). All washes were done in 0.1% PBST. Alexa Flour 488/546/647 conjugated secondary antibodies (life Technologies) were used in dilution of 1:500 for 1 h at room temperature. Mounting was done using ProLong Diamond Antifade Mountant with DAPI (P36962, Life Technologies). 3i CSU-W1 Spinning disk (objective 40×, 63× or 100×) microscope was used to obtain images. Acquired images were processed with ImageJ 1.8.0 software version (National Institute of Health, USA) and Adobe Photoshop.

### Histology

Testis and epididymis from *Smg6-*cKO and control mice were collected and directly fixed in 4% paraformaldehyde (PFA) or in Bouin fixative overnight at room temperature in gentle rotation. Tissues were dehydrated in a series of ethanol washes as described above and embedded in paraffin and cut to sections. Testis were stained with periodic acid-Schiff (PAS) and epididymis with hematoxylin–eosin (HE) according to standard protocols. Images were acquired with the microscope slide scanner Pannoramic P1000 (3D Histech) for bright field imaging with 40× objective.

### Isolation of the chromatoid bodies

CBs were isolated according to Meikar *et al.* with some modifications. Germ cells were released from three testes of adult control mice or foyr testes from *Smg6-*cKO mice with 0.05% (w/v) collagenase (Worthington). The cells were filtered through a 100-μm cell strainer (BD Falcon), washed with Phosphate buffered saline (PBS), and fixed in 0.1% PFA solution (Electron Microscopy Sciences, USA) for 20 min at RT. The fixed cells were lysed by sonication (UCD-200, Diagenode) 6 × 30 sec intervals with medium settings in 1.5 ml of RIPA buffer (50 mM Tris–HCl at pH 7.5, 1% NP-40, 0.5% w/v sodium deoxycholate, 0.05% w/v sodium dodecyl sulfate, 1 mM EDTA, 150 mM NaCl, 1× complete mini mix (Roche), 0.2 mM PMSF, and 1 mM DTT). The CB-enriched pellet fraction was separated by centrifugation at 300 × g for 10 min, resuspended in RIPA buffer, sonicated for an additional 2 × 30 sec using medium settings. Resulting lysates were equally divided and the CBs were immunoprecipitated using Dynabead Protein G (Invitrogen) with anti-DDX4 or anti-rabbit IgG (negative control) O/N at 4°C. For western blotting, samples from each isolation step (lysate of cross-linked cells; supernatant after low-speed centrifugation; CB-containing pellet fraction; CBs isolated by anti-DDX4 IP; and control IP using rabbit IgG) were diluted in Laemmli buffer and incubated 5 min at 95°C before loading them on the gel. For RNA-seq crosslinks of the isolated CBs were reversed by incubation at 70°C for 45 min.

### Immunoprecipitation

Three testes from adult mice were collected in 1.7 ml of isotonic nondenaturing lysis buffer (150 mM NaCl, 5 mM EDTA, 50 mM Tris–HCl, pH 8.0, 1% Triton X-100, 1× complete mini mix [Roche, 4693124001], 0.2 mM PMSF and 1 mM DTT). Samples were homogenized with TissueLyser LT (Qiagen) homogenizer for 90 sec and kept on ice for 30 min. Lysates were cleared by centrifugation at 14 000 × g for 10 min. Supernatant was divided to equally three parts, pre-cleared using 10 μl of Dynabead Protein G and incubated with either anti-SMG6, anti-SMG7 or rabbit IgG at 4°C overnight. Protein complexes were immunoprecipitated using 30 μl of Dynabead Protein G for 1 h RT. The Dynabeads–antibody–antigen complexes were washed 3 times in 1 ml of the non-denaturing lysis buffer.

### LC–ESI-MS/MS Analysis

Dynabeads–antibody–antigen complexes from IP with SMG6/SMG7/IgG or CB-IP/IgG were washed with 3 × 1 ml of Tris–HCl (pH 8.0). The LC–ESI-MS/MS analyses were performed on a nanoflow HPLC system (Easy-nLC1200, Thermo Fisher Scientific) coupled to the Orbitrap Fusion Lumos mass spectrometer (Thermo Fisher Scientific, Bremen, Germany) equipped with a nano-electrospray ionization source. Peptides were first loaded on a trapping column and subsequently separated inline on a 15 cm C18 column (75 μm × 15 cm, ReproSil-Pur 5 μm 200 Å C18-AQ, Dr Maisch HPLC GmbH, Ammerbuch-Entringen, Germany). The mobile phase consisted of water with 0.1% formic acid (solvent A) or acetonitrile/water (80:20 (v/v)) with 0.1% formic acid (solvent B). A linear 30 min gradient from 8% to 39% B was used to eluate peptides. MS data was acquired automatically by using Thermo Xcalibur 4.1 software (Thermo Fisher Scientific). An information dependent acquisition method consisted of an Orbitrap MS survey scan of mass range 300–1750 *m*/*z* followed by HCD fragmentation for most intense peptide ions.

### Generation of germ cell-specific *Smg6* conditional knockout mice (*Smg6*-cKO)

The genetic background of all the mice used in this study was mixed background with C57Bl/6J and SV129. The targeting construct for the generation of the *Smg6* conditional knockout (PG00253_Z_1_B03) was purchased from the International Mouse Phenotyping Consortium, and further verified by restriction enzyme digestion and sequencing. G4 embryonic stem cells (ES, derived from 129S6/C57BL/6Ncr mice) were cultured on neomycin-resistant primary embryonic fibroblast feeder layers and 10^7^ cells were electroporated with *Asi*SI linearized targeting construct. Cells were plated on 100 mm culture dishes with G418 (300 μg/ml; Sigma) after electroporation, and colonies were picked up after 7–9 days of selection, and grown on 96-well plates. In order to delete *Neo* cassette in the targeted ES cells, the cells were re-electroporated with plasmid pCAGGS-Cre and plated on 100 mm culture dishes. Colonies were picked up after 3–5 days of growth and grown on 96-well plates. Three targeted ES clones and several ES clones with right *Neo* deletion were determined by LR-PCR and PCR, and were further confirmed by sequencing. Two of the right targeted ES clones with *Neo* deletion were used for blastocyst injection in order to generate chimeras. Male chimeras from both line were bred with wild-type females to test the germline transmission. To achieve selective inactivation of *Smg6* in early postnatal germ cells, transgenic *Ngn3Cre* male mice (35–37) were mated with homozygous *Smg6* floxed females, *Smg6*(fx/fx) in order to generate *Smg6*(fx/wt);*Ngn3Cre*+ mice. *Smg6*(fx/wt);*Ngn3Cre*+ heterozygous males were then crossed with *Smg6*(fx/fx) females to produce *Smg6*(fx/fx);*Ngn3Cre* + cKO mice, as well as *Smg6*(fx/fx);*Ngn3Cre*– and *Smg6*(fx/wt);*Ngn3Cre*– littermates that were used as controls in all experiments. Genotyping of *Ngn3Cre* transgene was performed as reported before (35–37), and floxed *Smg6* allele was genotyped using specific primers flanking the flox site (Supplementary Table S7). The phenotype of *Smg6* depleted male mice was confirmed from mice derived from both clones.

### TUNEL assay

Testis from adult *Smg6-*cKO or control mice were collected and directly fixed in 4% PFA overnight at room temperature and embedded in paraffin. Paraffin-embedded testis sections were deparaffinized by incubation 3 × 3 min in xylene, 2 × 2 min in 100% ethanol, 2 × 2 min in 96% ethanol, 2 × 2 min in 70% ethanol and then washed in TBS (Tris-buffered saline, pH 7.5) 1 × 5 min. Sections were subjected to antigen retrieval in sodium citrate solution as described above. Slides were washed 3 × 5 min in TBS, incubated 10 min in 100 mM NH_4_Cl and TBS washes repeated. Slides were incubated for 1 h at 37 °C in humidified conditions with TUNEL (TdT-mediated dUTP nick end labeling) mixture: TdT buffer with terminal transferase (03333566001, Roche) 1U/μl, CoCl_2_, and 1 μM biotin-16-dUTP (11093070910, Roche) in MQ. Positive control sections were incubated with DNAase I, grade I (30 U/ml DNAase1 Invitrogen) for 30 min at 37 °C and negative control sections were incubated without terminal transferase (03333566001, Roche). The reaction was ended by incubating slides for 15 min RT with 300 mM NaCl, 30 mM NaCitrate in milliQ, and washed 4 × 5 min with TBS. Slides were blocked, incubated with specific antibodies, PNA and secondary antibodies, and mounted as described above. 3i CSU-W1 Spinning disk (objective 40x) microscope and Pannoramic Midi Fluorescence slide scanner (3D Histech) were used for obtaining images. Acquired images were processed as described above.

### Electron microscopy

Testis samples were fixed in 5% glutaraldehyde and treated with a potassium ferrocyanide-osmium fixative. The samples were embedded in epoxy resin (Glycidether 100, Merck), sectioned, post-stained with 5% uranyl acetate and 5% lead citrate, and visualized on a JEOL 1400 Plus transmission electron microscope (JEOL Ltd, Tokyo, Japan).

### Antibodies

Primary antibodies used in this study were: DDX25 (sc-51271) and Hiwi (sc-22685) from Santa Cruz Biotechnology; DDX4 (ab13840), EIF4A3 (ab32485), and SMG6 (ab87539) from Abcam; PIWIL1 (G82) from Cell Signaling Technology; FYCO1 (HPA035526 and SAB1400697) from Sigma-Aldrich; GAPDH (5G4) from HyTest; γH2AX antibody (05-636) and PIWIL2 clone 13E-3 (MABE363) from Millipore; SMG7 (A302-170A) from Bethyl Laboratories and hVasa (AF2030-SP) from R&D systems. Secondary antibodies conjugated with Alexa Fluor 488, 546, and 647 made in donkey were from Thermo Fisher Scientific (A-21202, A-21206, A-11055, A10036, A10040, A-11056, A-21203, A-21207, A-11058, A-31571, A-31573, A-21447). HRP-linked anti-rabbit IgG (NA934) and HRP-linked anti-mouse IgG (NA031) were from GE Healthcare Life Sciences.

### Isolation of germ cells

Round spermatids and spermatocytes were isolated from 3 to 6 testis from adult *Smg6-*cKO and control mice using our published protocol (38). Briefly, testes were de-capsulated and digested using consecutive collagenase IV (C5138, Sigma) and DNase I (DN25, Sigma-Aldrich) supplemented trypsin digestions (LS003703, Worthington) in 1× KREBS buffer (prepared as described in (38)). Cell suspension was washed with 1× KREBS filtered with 100 μm filter and loaded on a ready prepared ice-cold BSA gradient buffer. After 2 h of sedimentation, cells were collected, washed, and purity of fractions verified with DAPI staining.

### Seminiferous tubule cultures and Click reaction after EU labeling

Mouse testes were decapsulated in Dulbecco's modified Eagle's medium/nutrient mixture F-12 Ham (D8437, Sigma). Segments of the seminiferous epithelial cycle representing stages VII–VIII of spermatogenesis were cut as previously described (39,40). The isolated pieces of tubules were incubated on glass slides in 30 μl of medium supplemented with 1 mM ethynyl uridine (EU) at 34°C for 10 h in a highly humidified atmosphere containing 5% CO_2_. Spermatogenic cells were spread out of the cultured seminiferous tubules to monolayers using the squash technique (39,40), snap-frozen in liquid nitrogen, and fixed in 96% EtOH for 3 min. The nascent RNA was visualized using the Click-iT RNA Alexa Fluor 488 Imaging Kit (Molecular Probes, Invitrogen, C10329). Mounting was done using ProLong Diamond Antifade Mountant with DAPI (P36962, Life Technologies). 3i CSU-W1 Spinning disk (objective 40×, 63× or 100×) microscope was used for obtaining images. Acquired images were processed with ImageJ software version 1.8.0 (National Institute of Health, USA) and Adobe Photoshop.

### RNA extraction and detection of small RNAs

RNA was extracted from 24 dpp testes, enriched fractions of pachytene spermatocytes and round spermatids or isolated CBs with the Trisure reagent (Bioline) using standard protocols. Isolated RNA was analyzed using a NanoDrop (Thermo Scientific) and Bioanalyzer (Agilent). For the RNA profile of *Smg6-*cKO and control 24 dpp testes and CBs, the isolated RNA was separated in 10%–15% denaturing urea-polyacrylamide or 1–2% agarose gels, post-stained with SYBR Gold (Invitrogen), and visualized using Chemidoc Imaging (BioRad).

### Germ cell RNA sequencing and analysis

Three biological replicate samples from isolated round spermatids and spermatocytes from adult *Smg6-*cKO and control mice were submitted for RNA sequencing (Finnish Functional Genomics Centre, Turku Bioscience). Libraries were constructed with Illumina TruSeq Stranded mRNA polyA library preparation kit was performed with HiSeq3000 (read length 2 × 75 bp) sequencing system (Illumina, San Diego, CA, USA).

- DE analysis of genes, transposable elements, and piRNA precursors.

For gene-level DE analysis, reads were trimmed off adapter contents using cutadapt (v2.8) and mapped to the mouse genome (Ensembl: Grcm38) using STAR (v2.7.3a). Then, reads were assigned and counted using featureCounts (v2.0.0) against reference gtf file (Ensembl: Grcm38) in paired-end mode. First, raw read counts were filtered in order to keep only genes with 10 or more counts in at least three individual samples among six samples from a given cell type (3 × control and 3 × cKO). For transposable elements, reads were assigned and counted using TEtranscripts in reverse stranded mode (v2.2.1) (41) against reference genic and repeats gtf files (Ensembl: Grcm38). For piRNA precursor transcripts, reads were assigned and counted against the gtf file containing the 214 piRNA precursors (20) using featureCounts. Raw count reads were filtered to keep only genes with at least 10 reads across all samples *(Smg6-*cKO and control). Raw count reads were normalized and differential expression calculated using DESeq2 in R (v4.1.3). For transcript-level DE analysis, the procedure was similar than for gene-level except raw read counts were filtered in order to keep only transcripts with 20 or more counts in at least three individual samples. GO plots were generated using clusterProfiler. Expression clustering was based on normalized counts extracted from each samples using DESeq2. The genes were filtered according to differential expression analysis (DEA) and further normalized counts were scaled and averaged to finally use hclust() function in R for hierarchial clustering.

- Analysis of NMD-inducing features and stability

For analysis of NMD-inducing features, sequences were obtained using the UCSC Table Browser. To identify putative NMD features, we used a custom program developed by the laboratory that was previously published (42,43). Only Ensembl-defined transcripts harboring both 5′UTR and 3′UTR regions were considered for analysis. To find downstream exon junctions (dEJs), exon junction positions for each transcript were identified and dEJs were defined as EJs ≥50 nt from the PTC. The 5′ UTR sequences identified through the annotation process were used to scan for upstream open reading frames (uORFs) that contained particular nucleotides from the Kozak consensus sequence critical for initiating translation. In particular, we only considered uORFs ≥ 30 nt long that had a purine at the −3 position or a guanine at the +1 position (relative to the A in the AUG initiation codon [+1]). To reduce the probability of identifying uORFs that can re-initiate translation (and thus escape NMD), one additional criterion is that the uORF must not contain the main open reading frame. To infer the RNA stability, we used the REMBRANDTS program (44) following the tutorial (https://github.com/csglab/REMBRANDTS).

- Analysis of 3′UTRs

Transcripts were assembled using Cufflinks v2.2.1 for all samples individually. Cuffmerge was used to merge transcripts assemblies for all samples (control + *Smg6*-cKO). 3′UTR genomic locations were retained from cuffmerge output. A new gtf file was produced containing 3′UTR locations from assembled transcripts. Subsequently, a differential expression analysis was run using Cuffdiff in order to identify lowly expressed 3′UTR transcripts and filter merged transcripts from *Smg6*-cKO and control in the next step. In parallel, Cuffmerge was used to merge transcripts assemblies from control and *Smg6*-cKO samples separately. Merged transcripts from *Smg6*-cKO and control were trimmed to keep only 3′UTR locations and transcripts ≥1 FPKM from the previous step.

- Transcriptome comparison between *Smg6-*cKO and *Piwil1*-KO

The datasets for *Piwil1*-KO early round spermatids and late spermatocytes (GSE42004), *Piwil1-*KO pachytene spermatocytes and round spermatids (GSE64138) were downloaded from the NCBI GEO database. Reads were trimmed off adapter contents and low-quality bases were cut using trimmomatic (v0.39). The clean reads were mapped to the mouse genome (Ensembl: Mus_musculus.GRCm38.101) using STAR (v2.7.1a). Then, the reads were assigned and counted using featureCounts in R after mapping. Raw reads were filtered to keep genes with at least 10 reads found in the replicate samples. Raw reads were normalized and differential expression calculated using DESeq2. The piRNA targets were predicted using GTBuster without filtering ‘–distance-from-canonical-cut-site’ (https://github.com/weng-lab/GTBuster) and NCBI-BLAST. The piRNA species in round spermatids with normalized read counts ≥ 1 were kept. piRNA target sites were predicted at the transcript-level, and the potential piRNA targeted transcripts were then combined to genes according to their locations. The putative piRNA targets were identified based on two rules: (i) perfect pairing with the seed sequence at g2–g7 of the piRNA molecule, and (ii) varying number of additional base pairing (8, 10, 12 or 14 matches) after the seed sequence at g8–g21.

### Germ cell small RNA sequencing and analysis

The same spermatocyte and round spermatid samples used for RNA sequencing were subjected to small-RNA-seq using QIAseq miRNA library preparation kit and HiSeq2500 Rapid run sequencing platform (1 × 75 bp). SPORTS1.1 (45) was used to map small RNA reads successively rsRNA, miRNA, and tsRNA sequences extracted from mm10 UCSC genome files originating from rRNAdb, miRBase (v.22), and GtRNAdb (v.2.0) using SPORTS default settings. Using a pre-compiled Perl script from SPORTS1.1, the original locations of all sequences were identified and analyzed whether they originated from 5′ end, 3′ end or 3′ CCA end of tRNA. Then, reads mapping to rsRNA, miRNA, and tsRNA sequences were extracted from SPORTS output text file using R. Repeats from repeatMasker and piRNA precursor locations (20) were mapped to the mouse genome (UCSC: mm10) using HISAT2 (v2.1.0); then, reads were assigned and counted using featureCounts (v2.0.0) against reference gtf files. Raw read counts were filtered to keep only genes with at least one occurrence found per sample. FPM (fragment per million) was calculated after raw counts normalization using SPORTS output in R. Raw read counts were normalized and differential expression calculated using DESeq2.

### Chromatoid body RNA sequencing and analysis

Three *Smg6-*cKO and four control CB RNA samples were submitted for total RNA sequencing without ribosomal RNA removal (Novogene). Total RNA libraries were prepared by NEBNext® Ultra™ Directional RNA Library Prep Kit without fragmentation. Sequencing was performed with NovaSeq6000 (PE150) platform (Illumina). Gene and transcript level DE analysis, as well as the analysis for NMD-inducing features were performed as for germ cell samples.

### RT-qPCR

RNA was extracted from isolated germ cells using TRIzol (Thermo Fisher Scientific), following manufacturers’ protocols. After DNase I treatment (AMPD1, Sigma) one microgram of extracted RNA was reversed transcribed using sensiFAST cDNA synthesis Kit (BIO-65054, Meridian bioscience). RT-qPCR was performed using SsoAdvanced Universal SYBR Green Supermix (Bio-Rad Laboratories, catalog no. 1725270), and expression data were normalized to housekeeping genes (Supplementary Table S7). Data was analyzed using JMP Pro and GraphPad Prism 9.

### Statistical analysis

Statistical analysis was performed in R 4.1.3 or GraphPad Prism 9.0.0.

## RESULTS

### SMG6 is regulated during spermatogenesis and specifically localizes to the CB

**Figure 1.**
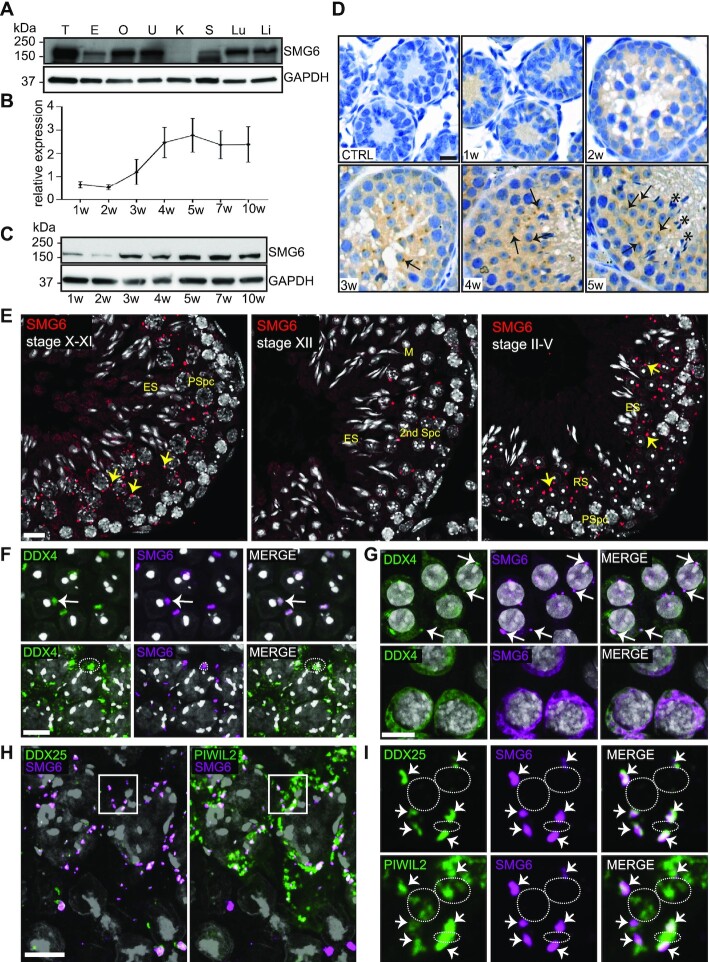
SMG6 localizes to the CB in round spermatids. (**A**) Western blotting of different tissues using anti-SMG6 antibody with anti-GAPDH as loading control. T, testis; E, epididymis; O, ovary; U, uterus; K, kidney; S, spleen; Lu, lung; Li, liver. (**B**) Relative expression of SMG6 during the first wave of spermatogenesis. Testis samples were collected from juvenile mice at different time points (1, 2, 3, 4 and 5 weeks) and adult mice (7 and 10 weeks). Two independent sample sets were included. Anti-SMG6 western blots were quantified with ImageJ software using anti-GAPDH for normalization. Error bars represent mean ± standard deviation (SD). (**C**) Representative western blot image of (B). (**D**) Immunohistochemistry of testis sections from 1, 2, 3, 4 and 5 weeks (w) old mice using anti-SMG6 antibody or no primary antibody (CTRL). Arrows point at selected examples of cytoplasmic SMG6 signal in round spermatids. Asterisks: elongating spermatid bundles. Scale bar: 5 μm. (**E**) IF of testis sections using anti-SMG6 antibody (red). PSpc, pachytene spermatocyte; RS, round spermatid; ES, elongating spermatid; M, meiotic metaphase plate; 2nd Spc, secondary spermatocyte. Arrows point at selected cytoplasmic granules. Scale bar: 10 μm. (**F**) Co-IF of SMG6 (magenta) and DDX4 (green) in round spermatids (upper panel, white arrow) and in late pachytene spermatocytes (lower panel, dashed circle). Scale bar: 10 μm. (**G**) Co-localization of SMG6 (magenta) with DDX4 (green) in human round spermatids (upper panel) and spermatocytes (lower panel). Arrows point at selected CBs. Scale bar: 10 μm. (**H**) SMG6 co-localizes with DDX25 and PIWIL2 to CB precursors in late pachytene spermatocytes (stage XI). SMG6 in magenta, DDX25 (left panel) and PIWIL2 (right panel) in green. Confocal 4-channel imaging was used to detect all three specific antibodies and DAPI in the same slides and artificial colors to each channel rendered afterwards using Adobe Photoshop. Scale bar: 10 μm. (**I**) The area indicated by a white box in panel H with higher magnification. Upper panel: co-localization of SMG6 with CB-specific DDX25. Lower panel: partial co-localization of SMG6 with PIWIL2. Co-localization points are marked by arrows. White dashed lines indicate examples of PIWIL2-positive (but DDX25- and SMG6- negative) IMCs. See also Supplementary Figure S1.

Our previous identification of SMG6 as a highly-enriched CB component (15) prompted us to further characterize its expression and function in male germ cells. We first examined SMG6 expression in different mouse tissues. Consistent with past studies showing that NMD factors are ubiquitously expressed (46), SMG6 was detected in all studied adult mouse tissues, though at a surprisingly low level in the kidney, and at a particularly prominent level in the testis (Figure 1A). In the juvenile mice during the first wave of spermatogenesis, at time points reflecting the appearance of specific spermatogenic cells, SMG6 is expressed at low level at postnatal week 1, when only somatic cells and spermatogonia are present, and at week 2, when spermatogonia have transitioned into spermatocytes. SMG6 expression increases at week 3, when spermatocytes convert into round spermatids, and further increases at week 4 and thereafter (Figure 1B, C). Immunohistochemistry confirmed SMG6 expression in round spermatids and demonstrated that SMG6 is primarily found in the cytoplasm (Figure 1D).

A detailed stage-specific immunofluorescence (IF) analysis demonstrated that SMG6 is first detectable at stage X–XI of the seminiferous epithelial cycle, with several cytoplasmic granules staining positive for SMG6 in late pachytene spermatocytes (Figure 1E). After the first meiotic division (stage XII tubules), secondary spermatocytes contain 1–3 cytoplasmic SMG6-positive granules, which condense into a single large granule in early round spermatids (stage II–V tubules), a finding consistent with the condensation of the CB at this stage (13). SMG6-positive granules remain in the cytoplasm of round spermatids during their subsequent steps of their differentiation (Figure 1E, Supplementary Figure S1). The SMG6 signal diminishes when spermatids begin to elongate (stage X–XI) and no signal is detected in condensed elongating spermatids (stage II–V) (Figure 1E, Supplementary Figure S1).

By co-localizing SMG6 with the well-established germ granule marker, DEAD box polypeptide 4 (DDX4), we confirmed the SMG6 positive foci represent CBs both in mouse (Figure 1F) and human round spermatids (Figure 1G), the latter demonstrating conservation. To study whether SMG6 also localizes to the IMC (13) we turned our attention to spermatocytes which contain both germ granule types. While both the CB and the IMC contain DDX4 (47), SMG6 only co-localized to some DDX4-positive cytoplasmic granules in late spermatocytes (Figure 1F, lower panel). Co-localization analysis with antibodies against DDX25, an ATP-dependent RNA helicase that localizes to CB precursors but not to the IMC, and PIWIL2, which localizes to both germ granule types (13), revealed that SMG6 signal overlapped with the DDX25/PIWIL2-positive CB precursors but not with PIWIL2-positive and DDX25-negative IMC (Figure. 1H,I, Supplementary Figure S1). Together, this data indicates that SMG6 is a CB loyal component from the early formation of CB precursors in pachytene spermatocytes to the fully mature CB in round spermatids.

### The localization and interactome of SMG6 and SMG7 in round spermatids differ

**Figure 2.**
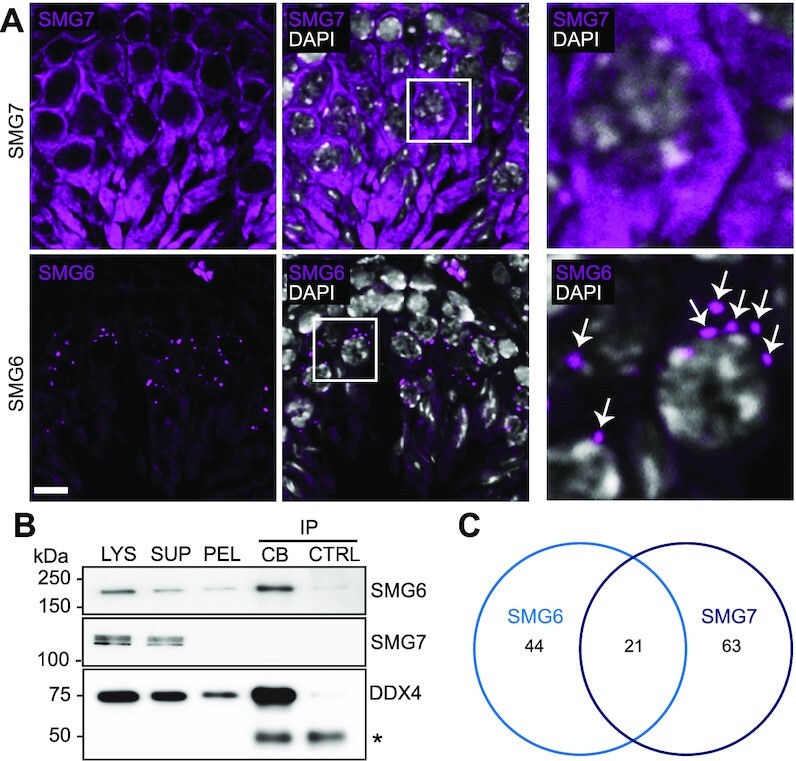
Characterization of SMG6 and SMG7 during spermatogenesis. (**A**) IF of adult mouse testis sections with either anti-SMG6 or anti-SMG7 antibody (both in magenta). Scale bar: 10 μm. The areas indicated by white boxes are shown in higher magnification in the right panel. White arrows indicate perinuclear SMG6-positive granules in spermatocytes. (**B**) Western blotting of isolated CBs using anti-SMG6 and anti-SMG7 antibodies. LYS, lysate of cross-linked cells; SUP, supernatant after low-speed centrifugation; PEL, CB-containing pellet fraction; CB-IP, CBs isolated by anti-DDX4 IP; CTRL, control IP using rabbit IgG. Anti-DDX4 Western blotting confirms the successful CB isolation. IgG heavy chain is indicated with an asterisk. Cross-linking causes a very weak background precipitation of SMG6 and DDX4 in the control IgG IP. (**C**) Venn diagram showing the unique SMG6 (44), unique SMG7 (63) and shared (21) interaction partners identified in the mass spectrometry analysis. See also Supplementary Figure S2 and Supplementary Table S1.

To address specificity, we examined whether other NMD factors are associated with the CB. We previously showed, using mass spectrometry, that purified CBs are not only enriched in SMG6, but also UPF1 and SMG1, as well as EJC components (15). IF analysis confirmed the CB-associated localization of SMG1 and UPF1 (Supplementary Figure S2A, B), and revealed another CB-associated NMD factor – UPF3B (Supplementary Figure S2C). Interestingly, unlike the other studied NMD factors (Supplementary Figure 2A–C), SMG7 exhibited diffuse distribution in the cytoplasm of germ cells (Figure 2A, Supplementary Figure S2D), with no specific enrichment to the CB. We used a DDX4-immunoprecipitation (IP)-based CB-isolation protocol developed by our group (16) to confirm the presence of SMG6, but not SMG7, in the CB fraction (Figure 2B). We also defined the SMG6 and SMG7 interactomes in the testis using IP followed by mass spectrometry. We identified 65 and 84 SMG6- and SMG7-interacting proteins, respectively, using a threshold of at least 1 peptide hit in at least two replicate IPs, with only 21 overlapping proteins (Figure 2C, Supplementary Figure S2E, Supplementary Table S1A). Among the SMG7-specific interacting proteins was SMG5, SMG7’s well established binding partner (33), as well as many ubiquitination pathway-associated proteins. The SMG6-interacting proteins included many CB-associated proteins, such as EIF4A3, EWSR1, TDRD3 and PCBP2 (Supplementary Table S1). Our results suggest that the SMG6- and SMG7-mediated NMD degradation pathways localize to different subcellular sites and act with different subsets of proteins in the male germline.

### *Smg6-*cKO male mice are infertile and lack spermatozoa

**Figure 3.**
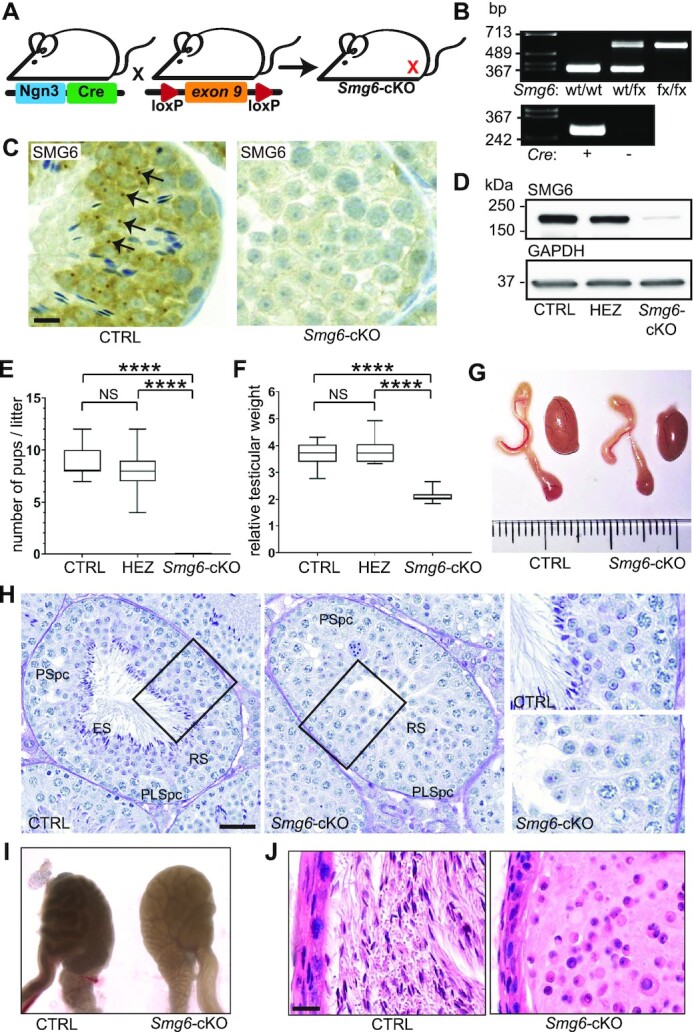
Germ cell-specific *Smg6-*cKO male mice are infertile. (**A**) To generate male germ cell-specific conditional *Smg6-*cKO mice, *Ngn3Cre* expressing mice were crossed with mice containing floxed exon 9 of *Smg6* gene. (**B**) Representative genotyping result of the floxed *Smg6* allele and transgenic *Ngn3Cre* construct. wt/wt, wild type; wt/fx, one floxed *Smg6* allele; fx/fx, two floxed *Smg6* alleles. (**C**) Validation of the knockout model by immunohistochemistry using anti-SMG6 antibody. SMG6-positive granules (indicated by black arrows) are not detectable in the *Smg6*-cKO testes. CTRL: *Smg6(fx/wt);Ngn3Cre*–, *Smg6-*cKO: *Smg6(fx/fx);Ngn3Cre+*. Scale bar: 10 μm. (**D**) Validation of the *Smg6* knockout by full testis western blotting with anti-SMG6 antibody. Anti-GAPDH antibody was used for normalization. HEZ: *Smg6(fx/wt);Ngn3Cre+ *. (**E**) Fertility analysis of CTRL (*Smg6(fx/wt);Ngn3Cre-*), HEZ (*Smg6(fx/wt);Ngn3Cre+*), and *Smg6-*cKO (*Smg6(fx/fx);Ngn3Cre+*) male mice. (**F**) Testicular size of CTRL (*n* = 9), HEZ (*n* = 8) and *Smg6-*cKO (*n* = 9) mice. Testicular weights were normalized to body weights. For (E) and (F), one way ANOVA with Tukeys's multiple comparisons test was used, ****P < 0.0001, NS = non-significant, mean ± SD indicated. **(G)** Representative macroscopic image of control and *Smg6-*cKO testis and epididymis. (**H**) Bouin-fixed paraffin-embedded testis sections stained with Periodic acid–Schiff (PAS). A representative example at the stage VII-VIII of the seminiferous epithelial cycle is shown. PLSpc, preleptotene spermatocyte; PSpc, pachytene spermatocyte; RS, round spermatid; ES, elongating spermatid. Scale bar: 50 μm. Areas indicated by black boxes are shown in higher magnification on the right. (**I**) CTRL and *Smg6-*cKO cauda epididymides under transillumination microscope. (**J**) Hematoxylin-eosin (HE) staining of PFA-fixed paraffin-embedded cauda epididymis sections. Scale bar: 20 μm. See also Supplementary Figures S2 and S3, and Supplementary Table S2.

To elucidate the physiological role of SMG6 during postnatal spermatogenesis, we generated a germ cell-specific *Smg6* conditional knockout (cKO) mouse line: *Smg6-*cKO. To achieve this, we first created a mouse line harboring loxP sites on either side of *Smg6* exon 9 (Figure 3A). To delete *Smg6* exon 9 specifically in early postnatal spermatogonia, we crossed these *Smg6*-floxed mice with a transgenic mouse line expressing the Cre recombinase under the control of the Neurogenin 3 (*Ngn3*)-promoter (Figure 3A,B) (35). Successful deletion of exon 9 from *Smg6* mRNA was confirmed using RNA-seq data of isolated germ cells (Supplementary Figure S2F). The other exons were normally expressed. Immunohistochemistry and Western blot analysis showed dramatically reduced level of the full-length SMG6 protein in *Smg6*-cKO testes (Figure 3C, D, Supplementary Figure S2G). A weak SMG6 band in the *Smg6-*cKO testis extract was detected (Figure 3D), likely due to SMG6 expression by CRE-negative cells, e.g. testicular somatic cells.

Breeding trials revealed that *Smg6-*cKO males produced no pups, even with prolonged co-caging with wt females (Figure 3E). In contrast, *Smg6-*cKO heterozygous males generated a comparable number of pups—with expected Mendelian ratios—when bred with wt females (Figure 3E). The testicular weight of *Smg6-*cKO males was significantly reduced compared to control mice (Figure 3F,G, Supplementary Table S2). *Smg6-*cKO heterozygotes had normal testicular weight (Figure 3F), consistent with their normal fertility (Figure 3E). A detailed histological examination showed that all 12 different stages of the seminiferous epithelial cycle (2) were identifiable in *Smg6-*cKO testes. However, the cellular composition of the epithelium was severely disrupted across these stages (Figure 3H, Supplementary Figure S3A). We detected spermatogonia, spermatocytes, and round spermatids in *Smg6-*cKO testis cross-sections, but no elongating spermatids or mature spermatozoa were observed (Figure 3H, Supplementary Figure S3A), suggesting that *Smg6-*cKO round spermatids failed to progress to form elongating spermatids. Consequently, no spermatozoa were detected in the *Smg6*-cKO epididymis; histological analysis of abnormally translucent epididymides of *Smg6-*cKO mice (Figure 3I) revealed a complete absence of mature spermatozoa in the cauda epididymis (Figure 3J) and other epididymal compartments that otherwise appeared histologically normal (Supplementary Figure S3B).

### Germ cell loss of SMG6 leads to spermatogenic arrest in haploid round spermatids

**Figure 4.**
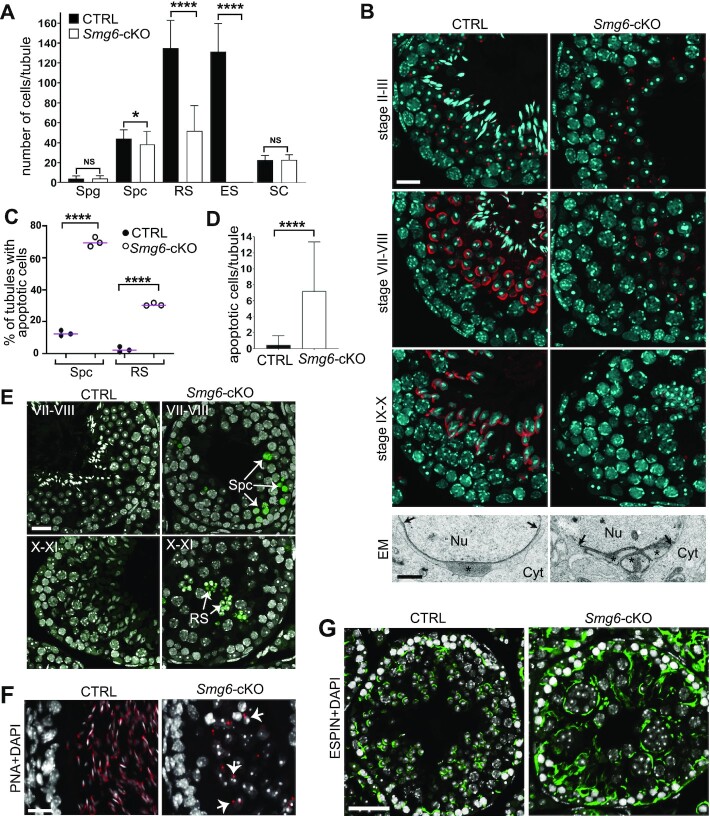
Spermatogenesis is arrested at round spermatid stage in *Smg6-*cKO mice. (**A**) Quantification of different cell types in CTRL and *Smg6-*cKO seminiferous epithelium. Quantified cell types included SOX3-positive spermatogonia (Spg), γH2AX-stained sex body-positive spermatocytes (Spc), round spermatids (RS) and elongating spermatids (ES) that were recognized on the basis of nuclear morphology and their position in the epithelium, and SOX9-positive Sertoli cells (SC). Cells were counted from a minimum of 70 tubules across three biological replicates per genotype. Error bars represent mean ± SD, and p-value from Mann-Whitney U-test, *P < 0.05, ****P < 0.0001, NS = non-significant. (**B**) PFA-fixed testis sections were stained with Rhodamine-conjugated PNA (red) to visualize acrosome and DAPI (blue) to visualize chromatin. Scale bar: 20 μm. The lower panel shows electron microscopy of the acrosomal region of round spermatids in control and *Smg6-*cKO mice. Nu, nucleus; Cyt, cytoplasm. Stars indicate the acrosome, which is fragmented into three separate granules in cKO. Arrows indicate the borders of the acrosomal region. Scale bar: 1 μm. (**C**) The percentage of tubules containing at least one apoptotic spermatocyte or round spermatids in a whole testis cross-section. At least 90 tubule cross-sections from each of the 3 CTRL and 3 *Smg6*-cKO mice were analyzed. Two-sided Fisher's exact test was used to demonstrate a significant association between CTRL and *Smg6*-cKO mice with the percentage of tubules with apoptotic cells, ****P-value < 0.0001. (**D**) Quantification of apoptotic cells per seminiferous tubule cross-section in CTRL and *Smg6-*cKO testes. Error bars represent mean ± SD, and *P*-value from Mann–Whitney *U*-test, ****P < 0.0001. (**E**) Representative TUNEL staining images of apoptotic spermatocytes (upper panel, stage VII-VIII) and round spermatids (lower panel, stage X–XI) on *Smg6-*cKO testis sections, together with matched stages from control testes without apoptotic cells. Scale bar: 20 μm. (**F**) PFA-fixed epididymides of control and *Smg6-*cKO mice were stained with PNA (red) and DAPI (white). Selected round spermatids with positive acrosome staining indicated by white arrows. Scale bar: 20 μm. (**G**) Testis sections were immunostained with anti-ESPIN antibody (green) to visualize apical ectoplasmic specializations at stage VIII-IX of the seminiferous epithelial cycle. Scale bar: 50 μm. See also Supplementary Figure S4.

To precisely elucidate the spermatogenic defect caused by the deletion of *Smg6*, we quantified specific testicular cell types in *Smg6-*cKO mice. *Smg6-*cKO testes had a dramatic reduction in round spermatids and only a modest reduction in pachytene spermatocytes, indicative of a round spermatid defect that initiates in spermatocytes (Figure 4A). As evidence for specificity, the number of undifferentiated spermatogonia (SOX3^+^ cells) and Sertoli cells (SOX9^+^ cells) was not significantly changed (Figure 4A, Supplementary Figure S4A, B). To elucidate what steps of round spermatid differentiation are most impacted by *Smg6* deletion, we examined acrosome biogenesis. We found that step 2–3 round spermatids, which have immature acrosomal granule, are present in *Smg6-*cKO mice (Figure 4B; stage II–III). However, we failed to find normal step 7–8 round spermatids, which are characterized by further developed acrosomes that spread over the nuclear envelope (Figure 4B; stage VII–VIII). At these stages, *Smg6-*cKO round spermatids had severe structural abnormalities, including acrosome fragmentation as detected by electron microscopy (Figure 4B: EM, Supplementary Figure S4C). No acrosomal staining was detected in stages IX–X, reflecting loss of spermatids as they transition from the round to the elongating phase (Figure 4B).

To determine whether apoptosis is responsible for the loss of germ cells in *Smg6-*cKO mice, we performed TUNEL analysis. This analysis showed that the percentage of tubules containing at least one apoptotic spermatocyte was >5-fold increased (from 13% in control to 70% in *Smg6-*cKO) and the percentage of tubules with at least one apoptotic round spermatid was 10-fold increased (from 3% to 31%) in *Smg6-*cKO mice (Figure 4C). Furthermore the overall number of apoptotic spermatocytes and round spermatids per tubule was dramatically increased (average 0.5 ± 1.1 in control versus 7.2 ± 6.1 in *Smg6-*cKO) (Figure 4D, E). During normal spermatogenesis, apoptotic spermatocytes are often observed at two ‘checkpoint stages’: mid-pachytene checkpoint at stage IV (48) and meiotic metaphase checkpoint at stage XII of the seminiferous tubule cycle (49). In *Smg6-*cKO testes, spermatocytes exhibited elevated apoptosis at these stages as well as those stages that normally have no apoptosis (Supplementary Figure S4D). Furthermore, while spermatid apoptosis at any stage is a rare event in wt testes, in *Smg6-*cKO apoptotic spermatids were frequently detected throughout the seminiferous epithelial cycle (Supplementary Figure S4D).

In addition to a dramatic increase in apoptotic germ cells in *Smg6-*cKO testes, we observed extensive sloughing of germ cells from the epithelium, as shown by the presence of round spermatid-appearing cells positive for acrosomal staining in the cauda epididymal lumen (Figure 4F). With ESPIN antibody that detects apical ectoplasmic specialization (50,51), we showed that these junctions between germ cells and Sertoli cells appear disorganized in *Smg6-*cKO testes (Figure 4G). This suggests that spermatids are prematurely released to the lumen due to their defective anchoring to the epithelium. Together, these results indicate that the loss of functional SMG6 in male germ cells results in disrupted haploid germ cell progression, widespread germ-cell apoptosis, and premature release of spermatids from the seminiferous epithelium. It remains to be determined whether the dramatic defect in haploid cell differentiation in *Smg6-*cKO mice originates from loss of SMG6 function in round spermatids, or is instead due to downstream effects of loss of SMG6 in meiotic cells.

### SMG6 loss causes transcriptome misregulation in spermatocytes and round spermatids

**Figure 5.**
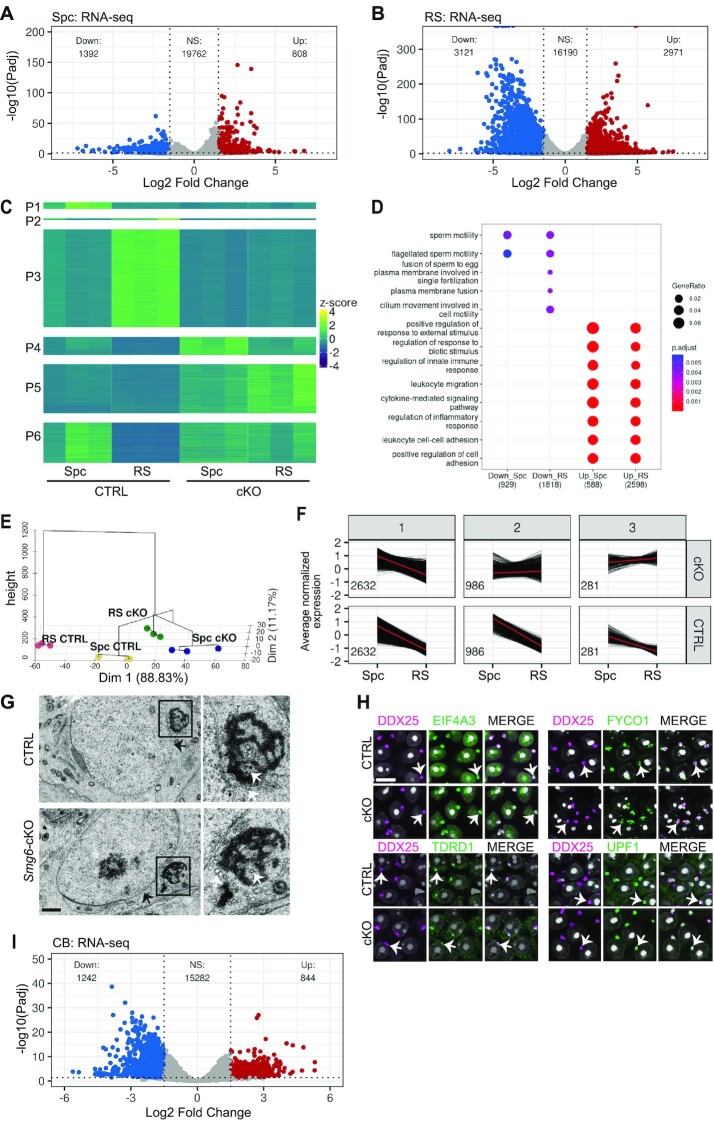
Deletion of *Smg6* induces transcriptome imbalance in germ cells. Volcano plots show differential expression of genes in spermatocytes (**A**) and round spermatids (**B**). (**C**) Heat-map visualizes the six expression patterns (P1-P6) after clustering according to the differential gene expression in CTRL Spc, CTRL RS, *Smg6-*cKO Spc and *Smg6-*cKO RS. (**D**) Biological Process GO term enrichment for down- and upregulated genes in *Smg6-*cKO versus CTRL Spc and RS. Top Biological Processes were selected for visualization based on adjusted *P*-values (gradient scale with red indicating the most significant value). GeneRatio: the amount of genes associated with the GO term divided by the total number of genes. Count: the number of genes associated to the GO term illustrated by a dot with a proportional size according to the number of associated genes found in our gene list. Plots were generated in R using clusterProfiler (v3.18.1). (**E**) Distribution and clustering of each group against each other (Spc CTRL, Spc cKO, RS CTRL, RS cKO) on a PCA plot, combined with a dendrogram using FactoMineR (v.2.4) in R. (**F**) Genes downregulated in CTRL RS versus Spc were partitioned into three expression patterns (1-3) according to their expression in *Smg6*-cKO germ cells. The number of differentially expressed genes for each expression pattern is indicated inside each panel. (**G**) Electron microscopy of the CB in CTRL and *Smg6-*cKO round spermatids. Scale bar: 1 μm. The CB indicated with black box is shown in higher magnification on the right panel. Black arrows indicate the nuclear membrane, white arrows indicate small vesicles located near or within the lobes of the CB. (**H**) PFA-fixed paraffin-embedded testis section were co-immunostained with anti-DDX25 (magenta) together with anti-EIF4A3, anti-FYCO1, anti-UPF1 or anti-TDRD1 (green). DAPI stains nuclei (white). White arrows indicate one selected CB in each figure. Scale bar: 10 μm. (**I**) Volcano plot to demonstrate differential expression of genes in CBs. See also Supplementary Figure S5 and Supplementary Tables S1 and S3.

Given that SMG6 is a critical component of the NMD pathway, this predicts that mRNAs targeted for decay by NMD would be dysregulated. To address this, we performed RNA sequencing (RNA-seq) on pachytene spermatocytes and round spermatids, both of which express high levels of SMG6 (Figure 1B-E). The purity of the cell fractions isolated by BSA-gradient velocity sedimentation (38), evaluated based on their DAPI staining, was ∼75% for pachytene spermatocytes and ∼80% for round spermatids (Supplementary Figure S5A). Differential expression (DE) analysis identified 808 and 1392 up- and down-regulated genes, respectively, in *Smg6-*cKO versus control spermatocytes, and 2971 and 3121 up- and down-regulated genes in round spermatids (Log2FC ≥ 1.5 or ≤–1.5, *P*_adj_ ≤ 0.05) (Figure 5A, B, Supplementary Table S3A, B). The upregulated genes are candidates to encode NMD target RNAs given that loss of SMG6 is expected to compromise NMD and lead to the upregulation of its targets. Most of the upregulated genes (88% in round spermatids) were protein-coding mRNAs; the rest corresponded to pseudogenes and other types of non-coding RNAs (Supplementary Table S3A, B). The misregulation of selected mRNAs was validated by RT-qPCR (Supplementary Figure S5B). Using a program that infers relative RNA stability based on pre-mRNA and steady-state mRNA levels (44), we found that 35% of the upregulated genes (with sufficient reads to be scored by the stability program) encode RNAs stabilized in both round spermatids (773/2214) and spermatocytes (166/475) (Supplementary Table S3D, E).

We partitioned the genes based on their expression patterns, and revealed that some genes were mis-regulated in both spermatocytes and round spermatids, while some were affected only in one cell type (Figure 5C). The six expression groups (P1–P6) included genes downregulated only in *Smg6-*cKO spermatocytes (P1; 212 genes), round spermatids (P3; 3073 genes) or both (P2; 60 genes), as well as genes upregulated in *Smg6-*cKO spermatocytes (P4; 571 genes), round spermatids (P6; 1254 genes) or both (P5: 1542 genes) (Figure 5C). GO analysis showed that the genes downregulated and upregulated in *Smg6*-cKO germ cells were associated with different biological processes (Figure 5D). Downregulated genes were significantly associated with functions related to fertilization and processes connected to the late steps of haploid differentiation such as flagellum biogenesis (Figure 5D). Many of these genes may be downregulated as a secondary consequence of arrested spermatogenesis. Upregulated genes were significantly enriched for biological processes not specifically linked to spermatogenesis, such as immune functions.

### The meiotic-to-postmeiotic transition program requires SMG6

Given that the expression of SMG6 peaks during the meiotic-to-postmeiotic transition (Figure 1F–I, Supplementary Figure S1) and that loss of SMG6 leads to strong defects in postmeiotic cells (Figure 3 and 4, Supplementary Figure S3), we considered the possibility that SMG6 might have a role in the gene program responsible for the meiotic-to-postmeiotic transition. Hierarchical clustering of the transcriptomes of *Smg6-*cKO and control spermatocytes (meiotic cells) and round spermatids (haploid postmeiotic cells) revealed that *Smg6-*cKO round spermatids clustered remarkably closely with control spermatocytes, suggesting that the loss of SMG6 greatly impedes the spermatocyte-to-round spermatid molecular progression program (Figure 5E). In further agreement, the number of differentially expressed genes in round spermatids compared to spermatocytes (log_2_FC ≥ 1.5 or ≤–1.5, *P*_adj_ ≤ 0.05) was much lower in *Smg6-*cKO (797 up and 1117 down) than in control (4411 up and 3899 down) mice (Supplementary Table S3F,G).

Clustering of the genes normally downregulated during the meiotic-to-postmeiotic transition (3899 genes) based on their expression in *Smg6*-cKO germ cells revealed that only a small portion was downregulated in *Smg6-*cKO rounds spermatids (Figure 5F, group 1), with the remaining genes either remained unchanged (group 2) or upregulated (group 3). GO term analysis showed that the genes that resisted downregulation in *Smg6-*cKO postmeiotic cells (groups 2 + 3) were associated with a diverse set of processes; e.g. transport, cell growth, and signaling (Supplementary Figure S5C). In contrast, the processes required for meiotic progression (e.g. chromosome segregation) appeared to be SMG6-independent since they were enriched in non-affected group 1 genes. Together, these results suggest that SMG6 promotes the degradation of a large set of meiotically expressed genes during the postmeiotic germ cell development, therefore contributing to the initiation of the haploid differentiation program.

### SMG6 influences the RNA composition of the CB

The enrichment of SMG6 and many other NMD factors in the CB (Supplementary Figure S2) suggests that the CB participates in SMG6-dependent NMD during the meiotic-to-postmeiotic transition. We thus investigated the effects of loss of SMG6 on the CB. Despite the reduced number of round spermatids and the halt in progression of those left in *Smg6*-cKO testes, the CBs in the remaining round spermatids appeared morphologically normal as assessed by electron microscopy. The network of dense material with interstices of irregular shapes and sizes intermingled with small vesicles was observed close to the nuclear membrane both in both *Smg6-*cKO and in the control (Figure 5G). Furthermore, well-established CB proteins (DDX25, EIF4A3, UPF1, FYCO1 and TDRD1) were readily detected in the *Smg6-*cKO CBs (Figure 5H), allowing us to purify *Smg6-*cKO CBs (16) for mass spectrometry (Supplementary Figure S5D). The integrity of the isolated CBs was verified by immunoblotting the CB extracts with an antibody against FYCO1, which localizes to CB-associated vesicles and thus co-precipitates with intact CBs (53) (Supplementary Figure S5D). *Smg6-*cKO CBs accumulated all the expected main CB components identified in our earlier study (15), including DDX4, DDX25, the PIWI proteins PIWIL1 and PIWIL2, and several Tudor domain-containing proteins (Supplementary Table S1B). Furthermore, the accumulation of RNA into the *Smg6-*cKO CB (15) was not measurably affected as, just like in the control, the nucleotide analog 5-ethynyl uridine (EU) labeled the *Smg6-*cKO CBs after a 10 h incubation in seminiferous tubule cultures (Supplementary Figure S5E).

Because the loss of functional SMG6 did not measurably disrupt CB formation, morphology, RNA import, or protein composition, we were able to study whether loss of SMG6 affects the RNA composition of the CB. Using high-throughput sequencing of isolated CBs from *Smg6-*cKO and control mice, we identified 844 significantly upregulated and 1242 downregulated genes (log_2_FC ≥ 1.5 or ≤–1.5, *P*_adj_ ≤ 0.05) in *Smg6-*cKO CBs (Figure 5I, Supplementary Table S3C). Given that the CB is primarily found in round spermatids, we compared mis-regulated genes in the CB and round spermatids. We found that the majority of both the upregulated (63% [532/844]) and the downregulated (79% [981/1242]) genes in the CB were also up- or downregulated in round spermatids, respectively (Supplementary Figure S5F). Thus, there is a clear overlap between the transcriptome changes in the *Smg6*-cKO CBs and round spermatids, suggesting that the CB has a central role in the SMG6-dependent transcriptome regulation. Of note, the CB and round spermatid datasets were generated using different library preparation pipelines and thus the overlap detected by our analysis may be an underestimate.

### Loss of SMG6 perturbs NMD and causes accumulation of transcripts with long 3′UTRs

NMD target RNAs can be tissue-specific (46) and are differentially regulated by different NMD factors (54). To address how the SMG6 target mRNAs we identified in the mouse germline correspond to NMD targets identified in other contexts, we looked for overlap with previously reported NMD target RNAs defined in a variety of mouse cell types and cell lines using different approaches, as compiled by Tan *et al.* from previous studies (43). The analysis revealed that 8% (49 of 587) and 17% (444 of 2621) of the protein-coding genes that are upregulated (log_2_FC ≥ 1.5, *P*_adj_ ≤ 0.05) in *Smg6-*cKO spermatocytes and round spermatids, respectively, are previously identified putative NMD target mRNAs (Supplementary Table S3A,B). Furthermore, 20% (143 of 717) of the protein-coding genes upregulated in *Smg6-*cKO CBs are encoded by previously identified putative NMD target genes defined by Tan *et al.* (43) (Supplementary Table S3C). Because the list of putative NMD target RNAs we used may include many false positives because they were identified only on the basis of only being upregulated (not necessarily stabilized) in NMD-deficient contexts (43), we did overlap analysis with 202 high-confidence targets genes that have been experimentally validated as encoding NMD target RNAs (34,42,61–63,43,46,55–60). This revealed that 38 of these 202 high-confidence NMD target genes were upregulated in *Smg6-*cKO round spermatids, and 16 were upregulated in *Smg6*-cKO CBs (Supplementary Table S3B,C), confirming that NMD is defective in *Smg6-*cKO germ cells. The relatively modest overlap raises the possibility that male germ cells have many novel NMD target mRNAs.

Several different ‘features’ in mRNA are known to target them for decay by NMD (33). To determine which of these might target mRNAs for decay in male germ cells, we examined the three most well-established NMD-inducing features: (i) an exon-exon junction downstream of the stop codon (dEJ), (ii) a short open reading frame upstream of the main ORF (uORF) and (iii) a long 3′ untranslated region (3′UTR) (33). We did not observe increased frequency of dEJs or uORFs in upregulated transcripts relative to either downregulated or unregulated transcripts in either spermatocytes or round spermatids (Supplementary Figure S5G, Supplementary Table S4A, B), suggesting that neither of these features commonly elicits SMG6-dependent NMD in these germ cell stages. However, the third feature – a long 3′UTR – was enriched in upregulated transcripts (32%) compared to downregulated or unregulated transcripts (19% and 27%, respectively) in *Smg6-*cKO round spermatids, but not in spermatocytes (Supplementary Figure S5G). We also examined NMD-inducing features frequency in transcripts that *resist* downregulation in *Smg6-*cKO postmeiotic cells (transcripts for the group 2 and 3 genes in Figure 5F). Remarkably, 41% of them contained long 3′UTRs (Supplementary Table S4D, E), providing further evidence that a long 3′UTR is a key feature targeting mRNAs for decay in round spermatids. The high frequency of long 3′UTRs in such mRNAs also suggests that NMD is directly responsible for the downregulation of many transcripts during the meiotic-to-postmeiotic transition.

**Figure 6.**
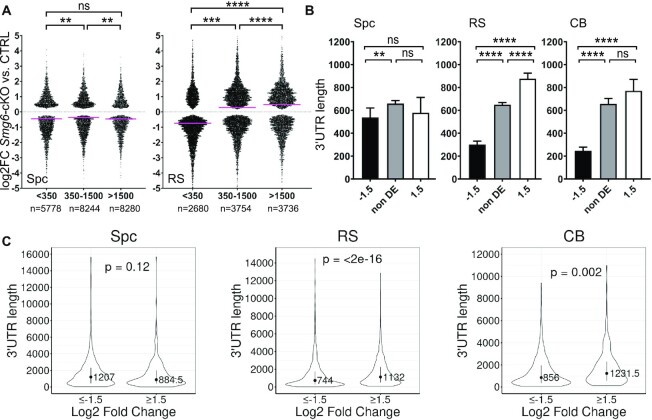
*Smg6* deletion affects the 3′UTR length distribution. (**A**) Swarm plot showing the distribution of fold change of transcripts in *Smg6*-cKO vs. CTRL for the significantly differentially expressed transcripts (y-axis; log2 fold change values) by 3′ UTR length (x-axis; length given in nucleotides). Transcripts were divided into three groups on the basis of their 3′UTR length: transcripts containing 3′UTR longer than 1500 nt, between 350 and 1500 nt, and shorter than 350 nt. Kruskal–Wallis test with Dunn's multiple comparisons was used to determine the significance values between groups (indicated with * above groups). Median values are indicated by pink lines (Spc > 1500: –0.4589, Spc 350–1500: –0.3597 and Spc < 350: –0.4494, RS > 1500: 0.4809, RS 350–1500: 0.2879 and RS < 350: –0.7401). (**B**) The length of 3′UTRs were plotted for downregulated (log_2_FC ≤ –1.5), non-differentially expressed (non DE, log_2_FC between –1.5 and 1.5) and upregulated (log_2_FC ≥ 1.5) transcripts in *Smg6*-cKO spermatocytes, round spermatids and CBs. A Kruskal-Wallis test and a Dunn post-hoc test was used to determine the difference between the median values of the groups (significance indicated with * above groups). Error bars represent median with 95% CI. (**C**) Violin plots showing the 3′UTR length for Cufflink-assembled DE transcripts (log_2_FC ≥ 1.5 and ≤ –1.5) in spermatocytes, round spermatids and CBs. Error bars represent median ± IQR, *P*-values from Wilcoxon-test. See also Supplementary Figure S5 and Supplementary Tables S4.

To further examine whether long 3′UTRs elicits NMD in round spermatids, we segregated transcripts into three groups according to their 3′UTR length (short: <350 nt, medium: 350–1500 nt, long: >1500 nt). Plotting them against their expression in *Smg6-*cKO vs. control round spermatids revealed that the median log2FC values of the long and medium 3′UTR groups (0.48 and 0.29, respectively) were significantly higher than that of the short 3′UTR group (–0.74) (Figure 6A). This correlation between 3′UTR length and steady-state RNA levels was observed only in *Smg6*-cKO round spermatids but not in spermatocytes (Figure 6A). There was also a striking difference in 3′UTR length between up- and downregulated transcripts in round spermatids, but not spermatocytes (Figure 6B). The 3′UTR length bias was also observed in the CB; the median 3′UTR length of upregulated transcripts in *Smg6-*cKO CBs was >3 times longer than downregulated transcripts (Figure 6B, Supplementary Table S4C). As an independent approach, we also analyzed 3′UTR lengths of transcripts assembled from our own datasets using the Cufflinks program, instead of relying on UTR information from other databases (as we did above). This analysis confirmed our finding that the median 3′UTR length is significantly increased in up- versus down-regulated transcripts in both round spermatids and CBs (Figure 6C). Together, these results strongly suggest that a NMD-inducing feature that commonly elicits RNA decay in round spermatids is a long 3′UTRs. Furthermore, our data raise the possibility that the CB is a specific site in round spermatids where long 3′UTR-mediated RNA decay occurs.

### SMG6 and PIWIL1 co-localize and interact in male germ cells

**Figure 7.**
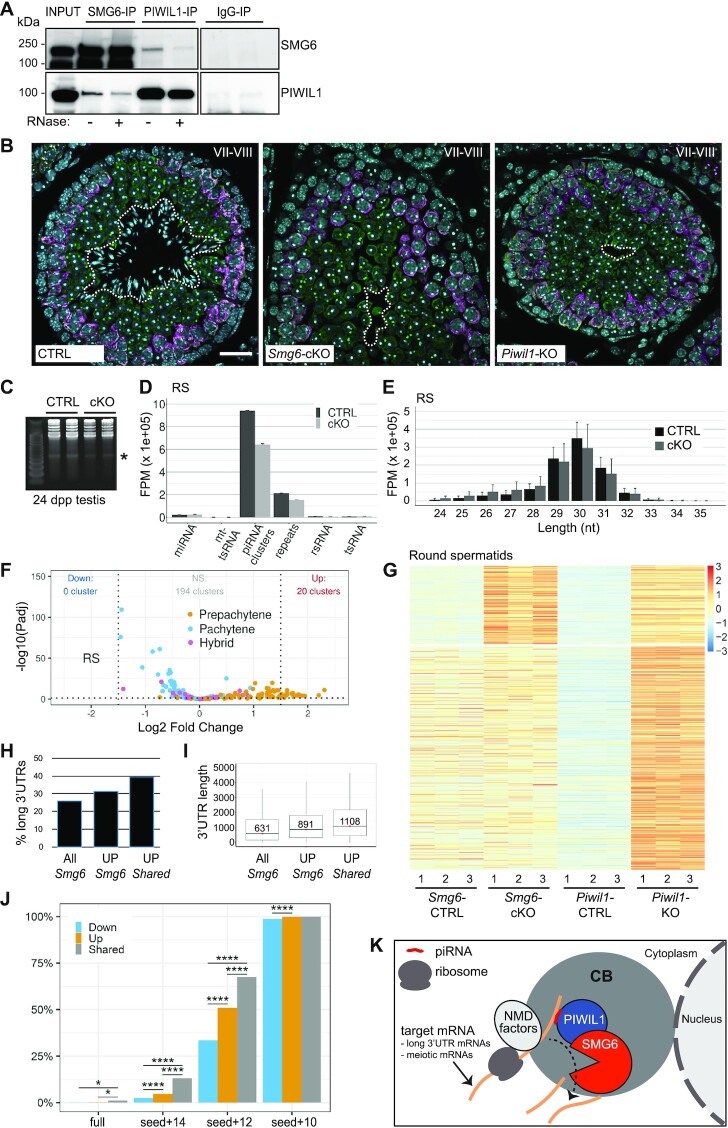
Interplay of SMG6 with the piRNA pathway. (**A**) Co-IP of SMG6 and PIWIL1 from mouse testis with or without RNase treatment (+/−), followed by western blotting with antibodies against SMG6 and PIWIL1. Rabbit IgG was used as a negative control for IP. (**B**) IF of PFA-fixed paraffin embedded testis sections from control (CTRL), *Smg6-*cKO and *Piwil1-*KO mice. Antibodies against DDX4 (green) and PIWIL2 (magenta) were used to visualize the layers of PIWIL2-positive pachytene spermatocytes and DDX4-positive CB-containing round spermatids at stage VII–VIII. DAPI stains the nuclei. Dashed white line indicates the border between the layers of round spermatids and elongating spermatids. Scale bar: 25 μm. (**C**) Visualization of piRNAs in 24 dpp *Smg6-*cKO testes. Total RNA from CTRL and *Smg6-*cKO testes was run into a denaturating 15% polyacrylamide gel and stained with SYBRCold. piRNA band (around 30 nucleotides) is indicated. (**D**) Distribution of small-RNA-seq reads in genomic regions corresponding to miRNAs, mitochondrial tsRNAs (mt-tsRNA), piRNA clusters, repeats, ribosomal RNAs (rsRNA), and tsRNAs in control and *Smg6-*cKO round spermatids. Error bars represent mean ± SD of three biological replicates. (**E**) Size distribution of piRNA-mapping reads in round spermatids. Raw counts were normalized in FPM. Error bars represent mean ± SD of three biological replicates. FPM values for (D) and (E) were calculated using a robust median ratio method using DESeq2 v1.30.0. (**F**) Differential expression of piRNA clusters (small-RNA-seq) in round spermatids. piRNA clusters are divided into pre-pachytene (84 clusters), hybrid (30 clusters), and pachytene (100 clusters) according to their abundance in juvenile testes from 10.5 to 17.5 dp (20). (**G**) Heatmap showing all genes upregulated in *Piwil1-*KO round spermatids clustered according to their expression pattern in *Smg6-*cKO germ cells. *Piwil1-*KO transcriptome data was downloaded from NCBI GEO database (GSE42004). (**H**) The proportion (%) of long 3′UTR-containing transcripts (3′UTR > 1500 nt) among all significant transcripts (All *Smg6*, 25.9% of 22302 transcripts; Padj ≤ 0.05), transcripts upregulated in *Smg6-*cKO round spermatids (UP *Smg6*, 31.4% of 5771 transcripts; *P*_adj_ ≤ 0.05, log_2_FC ≥ 1), and transcripts upregulated in both *Smg6-*cKO and *Piwil1-*KO round spermatids (UP *Shared*, 39.3% of 244 transcripts). (**I**) Box plots show the median values of 3′UTR length with the first and third quartiles for the same transcripts, whiskers show the range within 1.5 IQR (interquartile range). Outliers were removed for better visualization. (**J**) Percentage of genes with piRNA targeting sequenced among genes that are downregulated (Down) or upregulated (Up) in *Smg6*-cKO round spermatids, or upregulated in both *Smg6*-cKO and *Piwil1*-KO round spermatids (Shared). Pairing rules in target recognition: full = piRNA fully complementary to target mRNA, seed + 14, 12 or 10 = piRNA seed region fully complementary to target RNA plus additional 14, 12 or 10 nucleotide matches after the seed region. **P < 0.01; ****P < 0.0001, Fisher's exact test. (**K**) Model. NMD-recognized SMG6 target mRNAs are transported to the CB. piRNA-bound PIWIL1 participates in the recognition of SMG6-dependent NMD targets. See also Supplementary Figures S6 and S7, and Supplementary Tables S5 and S6.

Given that the CB is not only enriched for NMD pathway components but also piRNA pathway components, we considered the possibility that these two pathways are functionally connected. As support, the temporal expression and localization pattern of PIWIL1—the main pachytene piRNA-binding protein in round spermatids—is almost identical to that of the NMD factor SMG6 (Figure 1, Supplementary Figure S1 and Supplementary Figure S6). Like SMG6, PIWIL1 predominantly co-localizes with DDX25 in both CB precursors and mature CBs (Supplementary Figure S6A). Second, SMG6 and PIWIL1 form complexes in the mouse testes, as shown by co-IP analysis (Figure 7A). Their interaction appears to be at least partially mediated by RNA since RNase treatment weakened the signal (Figure 7A). The localization of PIWIL1 to the CB in round spermatids was not affected by the absence of SMG6 (Supplementary Figure S6B). SMG6 also retained enrichment in the CB in *Piwil1*-KO testes (64) (Supplementary Figure S6B). Therefore, their interaction is not required for the targeting of SMG6 and PIWIL1 to the CB. Further supporting a functional connection between SMG6 and PIWIL1, the testes defects in *Smg6-*cKO and *Piwil1-*KO mice were almost identical. In both knockout models, spermatogenesis is arrested at the round spermatid phase (Figure 7B, Supplementary Figure S6C, D) (64). In both knockout mice, round spermatids are detectable at stages I-VIII of the seminiferous epithelial cycle, but spermatids disappear from seminiferous epithelia at stage IX, the stage at which round spermatids transition to elongating spermatids (Figure 7B, Supplementary Figure S6C, D).

### Pre-pachytene piRNA clusters are upregulated in *Smg6*-cKO rounds spermatids

To further address the potential functional co-operation of SMG6 with the piRNA pathway, we first examined whether loss of SMG6 affects the piRNA production. Gel electrophoresis analysis of *Smg6*-cKO and control testes RNA showed that pachytene piRNAs are present in *Smg6*-cKO testes (Figure 7C). To study the effects of *Smg6* deletion on piRNA population in more detail, we performed small-RNA-seq analysis. The majority of reads mapped to the previously identified 214 piRNA clusters (20) in both *Smg6-*cKO and control round spermatids (Figure 7D) and spermatocytes (Supplementary Figure S7A). The size distribution of piRNAs was not affected in *Smg6*-cKO germ cells (Figure 7E, Supplementary Figure S7B), suggesting that there are no defects in the processing of mature piRNAs.

To determine whether the expression of particular piRNA clusters is affected in *Smg6*-cKO germ cells, we mapped the small-RNA-seq reads separately to piRNA clusters classified according to their temporal expression patterns during spermatogenesis: ‘pre-pachytene’ (84 clusters, expressed already at 10.5 dpp), ‘pachytene’ (100 clusters, expression emerges at 12.5 dpp), and ‘hybrid’ (30 clusters, this class shares expression characteristics with both pre-pachytene and pachytene clusters) (20). Analysis of these three classes of piRNAs revealed a selective effect of the *Smg6* deletion on the pre-pachytene piRNA clusters, which tended to be more abundantly expressed in *Smg6*-cKO germ cells (Supplementary Figure S7C). In total, 20 pre-pachytene piRNA clusters were significantly upregulated in *Smg6*-cKO round spermatids (log_2_FC ≥ 1.5, *P*_adj_ ≤ 0.05) (Figure 7F, Supplementary Figure S7D, Supplementary Table S5). We also examined piRNA precursor levels from our long-RNA-seq datasets. While this showed misregulation of some piRNA precursors (Supplementary Figure S7E, F, Supplementary Table S6A–C), there was no correlation between the altered expression of precursors vs. mature piRNAs in *Smg6*-cKO germ cells (Supplementary Figure S7G, H). Thus, the changed levels of mature piRNAs in *Smg6-*cKO is unlikely to be explained by a piRNA processing defect.

### SMG6 and PIWIL1 regulate partially overlapping set of genes

Next, we examined whether *Smg6* deletion causes misregulation of transcripts known to be targeted by the piRNA pathway. We first determined the expression of transposable elements in *Smg6*-cKO germ cells. We found some misregulated transposable elements in *Smg6*-cKO spermatocytes, rounds spermatids and CBs, but no indications of major defects in transposon silencing (Supplementary Table S6D–F). We next assessed potential shared mRNA targets by comparing the *Smg6-*cKO and *Piwil1-*KO round spermatid transcriptomes, the latter of which we reprocessed using available datasets in public repositories. Although it is not clear if piRNAs are involved in the regulation of RNAs that are altered in *Piwil1*-KO, PIWIL1 is the main piRNA-binding protein in round spermatids (17,21), and the transcriptome defects are likely to reflect the defects in the piRNA pathway. We focused our analysis on genes that are upregulated as a consequence of either SMG6 or PIWIL1 deletion. Differential expression analysis identified 997 and 1282 significantly upregulated genes in *Piwil1-*KO round spermatids (*P*_adj_ ≤ 0.05, log_2_FC ≥ 1) in two independent datasets (GSE42004 and GSE64138, respectively). One-fourth of these *Piwil1-*KO upregulated genes were also upregulated in *Smg6-*cKO round spermatids (259/997 genes for GSE42004 and 311/1282 genes for GSE64138; Figure 7G shows the data for GSE42004). The overlap between *Piwil1*-KO upregulated genes (997) and *Smg6*-cKO upregulated genes (4812) was statistically significant (p = 4.9e-05, using Fisher's exact test with genomic background being all 23 024 genes detectably expressed in *Smg6*-cKO and *Piwil1*-KO round spermatids).

Given that long 3′UTRs are known to trigger NMD (33) and our evidence that this particular NMD-inducing feature is a major signal triggering NMD in round spermatids (Figure 6, Supplementary Figure S5), we next assessed whether this feature is shared with PIWIL1. In particular, we examined whether this NMD-inducing feature is enriched in transcripts regulated by *both* SMG6 and PIWIL1 (i.e. upregulated in both *Smg6*-cKO and *Piwil1*-KO round spermatids). We found that such ‘shared transcripts’ were not only enriched for long 3'UTRs, but they had a greater proportion of long 3′UTRs than did *Smg6-*cKO round spermatid upregulated transcripts (Figure 7H). Furthermore, the median length of 3′UTR was longer in these shared upregulated transcripts vs. upregulated transcripts in *Smg6-*cKO round spermatids (Figure 7I). Together, this data indicates that PIWIL1 participates with SMG6 in regulating transcripts with long 3′UTRs.

### piRNA targeting sites are enriched in SMG6-PIWIL1-regulated genes

To further examine whether SMG6 and the piRNA pathway collaborate, we determined whether SMG6-regulated RNAs are predicted to be targeted by piRNAs. We used different criteria for piRNA target identification: (i) full complementary between piRNA and its target RNA, or (ii) full complementarity in the seed region at g2–g7 plus 14, 12, 10 or 8 additional matches at g8–g21 after the seed region. We then analyzed these predicted piRNA-targeting sites in genes differentially expressed in *Smg6*-cKO round spermatids. Using stringent criteria (seed + 14 or 12 matches), we found the proportion of predicted piRNA targets among genes upregulated in *Smg6*-cKO round spermatids (*P*_adj_ ≤ 0.05, log_2_FC ≥ 1) was higher than for downregulated genes (4.7% versus 2.5% for seed + 14, and 51% versus 34% for seed + 12). Importantly, the proportion of predicted piRNA-targeted genes was dramatically higher for genes that were upregulated in *both Smg6*-cKO and *Piwil1*-KO round spermatids (13% for seed + 14, and 68% for seed + 12) (Figure 7J). Together, these computational data support the notion that the piRNA pathway participates in SMG6-mediated RNA regulation.

## DISCUSSION

The CB was first identified as a cytoplasmic body in germ cells over 100 years ago (65). The CB has since been shown to house many RNA regulatory proteins and a broad range of different RNA species, but its precise function in these cells has remained unclear (13,15). In this study, we began to fill these gaps in our knowledge by uncovering the functions of a highly CB-enriched factor—the endonuclease SMG6. We demonstrated that loss of SMG6 in spermatocytes and round spermatids leads to a complete block in spermatogenesis. Transcriptome profiling identified three main classes of misregulated transcripts that are candidates to be responsible. Class I mRNAs are transcribed from genes normally silenced when spermatocytes transition to form round spermatids. Class II RNAs are targeted for decay by NMD, which is consistent with the fact that SMG6 is the sole endonuclease in the NMD pathway (33). Class III mRNAs are also regulated by the piRNA pathway component PIWIL1, indicating a potential collaboration between SMG6 and piRNA pathway in developing germ cells. Of note, these three classes of SMG6-regulated RNAs have some overlap. For example, we found that many misregulated spermatocyte mRNAs (class I) are also NMD targets (class II). Together, our results support a model in which the CB serves as a central site for both SMG6 and PIWIL1 function, and highlight the CB as a subcellular platform that facilitates compartmentalization and coordination of RNA regulatory pathways in the male germline (Figure 7K).

Our discovery that loss of SMG6 causes pronounced defects in the gene expression program associated with the meiotic-to-postmeiotic transition is consistent with our finding that SMG6 expression peaks in late spermatocytes and round spermatids. Most notable was that over thousand meiotically-expressed genes failed to be downregulated after completion of meiosis in *Smg6*-cKO round spermatids, a defect which we speculate contributes to the failure of round spermatids to undergo differentiation. Interestingly, many of these meiotic mRNAs appear to be NMD target mRNAs, raising the possibility that NMD has a direct role in the spermatocyte-to-spermatid transition. Many of the genes resisting downregulation in *Smg6-*cKO round spermatids encode proteins involved in processes not expected to be associated with spermatogenesis, such as wound healing, gliogenesis, and osteoclast differentiation. The failure to eliminate transcripts that do not have a clear functional role in spermatogenesis raises the intriguing possibility that SMG6, and by implication, NMD, is critical for destabilizing mRNAs synthesized as a result of the ‘leaky transcription’ known to occur in spermatocytes (6,10). If these mRNAs are not destabilized, their gene products could initiate a differentiation program incompatible with the spermatid differentiation program, leading to abnormal haploid differentiation and germ cell death, as we observed to be the case in *Smg6-*cKO mice.

Our data indicates that long 3′UTRs drive SMG6-dependent NMD in developing germ cells. One line of evidence for this was the accumulation of long (>1500 nt) 3′UTR-harboring transcripts in *Smg6-*cKO round spermatids. In addition, the median 3′UTR length of transcripts upregulated in *Smg6-*cKO round spermatids was much longer than that of downregulated and non-regulated transcripts. Consistent with these findings, conditional loss of the NMD factor, UPF2, in male germ cells in mice was also found to upregulate transcripts with long 3′UTRs (66). Similarly, loss of the CB-scaffolding protein, TDRD6, was shown to preferentially upregulate long 3′UTR-containing transcripts (relative to those with medium or short 3′UTRs) in round spermatids (67). This upregulation of long 3′UTR transcripts in *Tdrd6*-null round spermatids may be a consequence of disrupted NMD in the CB, as the central NMD factor, UPF1, is no longer detectable in the CB of *Tdrd6*-null round spermatids (67). Interestingly, it is well established that spermatocytes and spermatids tend to have transcripts with shorter 3′UTRs compared to somatic cells (68,69). While this has been attributed to germ cell-specific early polyadenylation site usage (70), an alternative explanation is that mRNAs harboring long 3′ UTRs are more strongly degraded by NMD in germ cells than in somatic cells, thereby allowing a selective accumulation of mRNAs with short 3′ UTRs.

The best-established signal that elicits NMD in somatic cells is a translation termination codon followed by at least one downstream exon-exon junction (dEJ) (28). Intriguingly, meiotic and postmeiotic germ cells have been reported to inefficiently (or not) recognize this NMD-inducing signal (66,67,71). Consistent with this, we failed to observe an enrichment for dEJ-containing transcripts among upregulated transcripts (vs. downregulated or unregulated transcripts) in *Smg6-*cKO germ cells. However, we found that *Smg6-*cKO germ cells *do* upregulate many dEJ-containing transcripts, including well-known NMD substrates; *e.g. Rassf1*, *Ptch1*, *Slc38a6*, *Usp22*, *HnrnpI*, *Wdr8*2 and *Tsr2*. This suggests that SMG6 *does* degrade mRNAs marked with a dEJ in male germ cells (72,73). However, male germ cells may recognize this NMD-inducing feature less efficiently than do somatic cells.

Our results support a role for the CB as an important subcellular platform for the SMG6 function, but whether SMG6-dependent NMD occurs in the CB remains unclear. In support of the CB being an active site of NMD, many NMD factors in addition to SMG6 are highly enriched in the CB (Figure 1, Supplementary Figs. S1 and S2) (15,66,67). The CB has indeed been shown to be associated with translation, a process essential to trigger the NMD pathway (29,30,43,74). The CB contains ribosomal proteins and ribosomal RNAs, based on high-throughput analyses (15). Polysome-like structures have been observed in CB isolated from rat spermatids (75), and inhibition of protein synthesis dramatically increases the size of the CB in *Xenopus laevis* (76). Finally, the CB has a close physical association with the endoplasmic reticulum, where secreted and cell-surface proteins are translated (53,77). However, other evidence suggests that the CB harbors many translationally repressed mRNAs (16) that are predicted to escape NMD. Thus, the CB may instead be a ‘NMD refuge’ – a site for sequestering NMD factors away from the cytosol to confer NMD-dependent regulation.

The co-compartmentalization of NMD and pachytene piRNA pathway components in the same subcellular structure in germ cells is intriguing. The co-localization of these components in the CB has the potential to enable close communication between these two pathways, thereby raising the possibility of their functional co-operation. In principal, the CB could be a site of piRNA biogenesis, but this seems unlikely given that the main piRNA biogenesis factors associate with mitochondria membranes and mainly localize to the IMC, not the CB (13). In further support, our results showed that the overall production of piRNAs was not compromised by the loss of SMG6. Instead, we found that SMG6 loss causes misregulation of mature piRNAs from several pre-pachytene piRNA clusters. The mechanism responsible for this SMG6-dependent regulation of a subset of piRNAs remains to be determined.

The CB has been suggested to function as a site of piRNA-targeted transcriptome regulation on the basis of its harboring piRNAs, piRNA-binding PIWI proteins and a wide range of mRNAs and non-coding RNAs (13,15). However, there is no direct mechanistic evidence for this function. If indeed the CB functions in this manner, our results suggest that SMG6 may participate in this post-transcriptional program to promote spermatid differentiation. In support, we showed that SMG6 and PIWIL1 interact and are both required for progression through the same step of spermatogenesis. Furthermore, we found that SMG6 and PIWIL1 regulate a shared set of genes in germ cells at the same stage of development. Given that SMG6 is an endonuclease (52), it is plausible that SMG6 directly degrades many of these mRNAs.

We favor a model in which piRNA-bound PIWIL1 participates in the recognition of SMG6-dependent NMD targets (Figure 7K). This model is based on the studies in *Drosophila* showing that PIWI-loaded piRNAs can act as adhesive mRNA traps and use partial base-pairing to bind mRNAs in a relatively non-sequence-specific manner to capture mRNAs in the *Drosophila* germ plasm (78). We suggest that a similar scenario takes place in mouse round spermatids, with PIWIL1-bound piRNAs functioning in the recruitment of SMG6-regulated mRNAs. To support this model, we found that piRNA targeting sites were enriched among the mRNAs encoded by genes upregulated in *Smg6*-cKO round spermatids compared to downregulated genes, and they were even more enriched in mRNAs encoded by genes upregulated in both *Smg6*-cKO and *Piwil1*-KO germ cells. piRNA-targeted cleavage by PIWIL1 alone was shown to be intrinsically slow, and PIWIL1 requires an auxiliary factor GTSF1 that potentiates the cleavage (79). GTSF1 is not found in the CB proteome (Supplementary Table S1B), which could, according to our model, direct the piRNA-targeted RNAs to SMG6 cleavage instead of PIWIL1 cleavage. Given that NMD depends on translation (29–31), it is an interesting possibility that piRNA/SMG6 target recognition takes place at ribosomes. PIWIL1 and piRNAs have indeed been shown to associate with ribosomes (80), and it was reported that a subset of PIWIL1-bound piRNAs functions in translational control (25). SMG6 and PIWIL1 could therefore interact during translation termination when NMD targets are first recognized. Based on all these findings, our model suggests an intimate relationship between the CB, translation, NMD and piRNA pathway (Figure 7K), which enables their collaboration to promote haploid germ cell differentiation.

## DATA AVAILABILITY

RNA-seq data are deposited in Gene Expression Omnibus (GEO) under accession number GSE182518. The mass spectrometry proteomics data have been deposited to the ProteomeXchange Consortium via the PRIDE partner repository with the dataset identifier PXD037090.

## Supplementary Material

gkac900_Supplemental_Files
