# Supplementary material for: SMG6 localizes to the chromatoid body and shapes the male germ cell transcriptome to drive spermatogenesis
Source: Nucleic Acids Res. 2022 Oct 19;50(20):11470–91. doi: 10.1093/nar/gkac900 (PMC9723633; doi:10.1093/nar/gkac900)
Supplement: gkac900_Supplemental_Files [file gkac900_supplemental_files.zip › Lehtiniemi_Supplementary Data.pdf]

SUPPLEMENTARY DATA

SMG6 localizes to the chromatoid body and shapes the male germ cell transcriptome to drive spermatogenesis

Tiina Lehtiniemi[1], Matthieu Bourgery[1], Lin Ma[1], Ammar Ahmedani[1], Margareeta Mäkelä[1], Juho Asteljoki[1], Opeyemi Olotu[1], Samuli Laasanen[1], Fu-Ping Zhang[1,2,3], Kun Tan[4], Jennifer N. Chousal[4], Dana Burow[4], Satu Koskinen[5], Asta Laiho[5], Laura L. Elo[1,5], Frédéric Chalmel[6], Miles F. Wilkinson[4, 7] and Noora Kotaja[1, #]

[1] Institute of Biomedicine, Research Centre for Integrative Physiology and Pharmacology, University of Turku, Finland
[2] Turku Center for Disease Modeling, University of Turku, Turku, Finland
[3] GM-Unit, Helsinki Institute of Life Science, Faculty of Medicine, University of Helsinki
[4] Department of Obstetrics, Gynecology, and Reproductive Sciences, School of Medicine, University of California San Diego, La Jolla, CA 92093, USA
[5] Turku Bioscience Centre, University of Turku and Åbo Akademi University, Turku, Finland
[6] Univ Rennes, Inserm, EHESP, Irset (Institut de recherche en santé, environnement et travail) - UMR_S 1085, F-35000, Rennes, France
[7] Institute for Genomic Medicine (IGM), University of California, San Diego, La Jolla, CA 92093, USA

Supplementary Figures S1-S7

Supplementary Figure S1

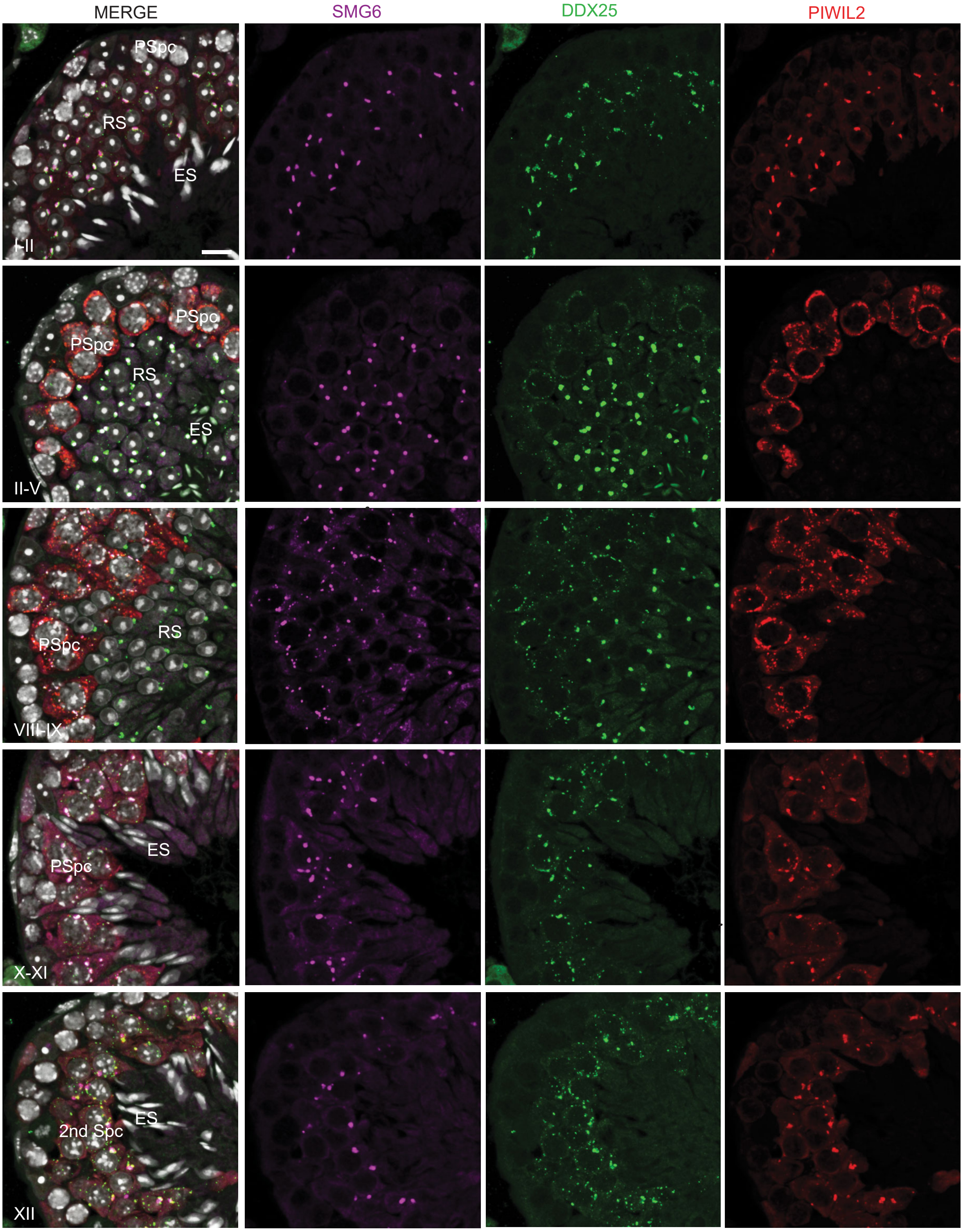

Supplementary Figure S1. Co-localization of SMG6 with DDX25 and PIWIL2. PFA-fixed paraffin-embedded testis sections were immunostained with SMG6 (magenta), DDX25 (green) and PIWIL2 (red) antibodies. PIWIL2-positive IMC is prominent in the cytoplasm of early and mid-pachytene spermatocytes (PSpc, stages II-V and VIII-IX). DDX25/PIWIL2-positive CB precursors appear in stage VIII-IX pachytene spermatocytes, and the IMC begins disintegrating. Secondary spermatocytes (2nd Spc, stage XII) contain 1-4 CB fragments, and after meiosis, they fuse to one single CB in round spermatids (RS, stages I-II). PIWIL2 is present only in early CBs at stages I-II. DDX25 labels the CB during all steps of round spermatid differentiation (stages I-II, II-V, IX) and finally disappears in elongating spermatids (ES). Scale bar: 10 µm.

Supplementary Figure S2

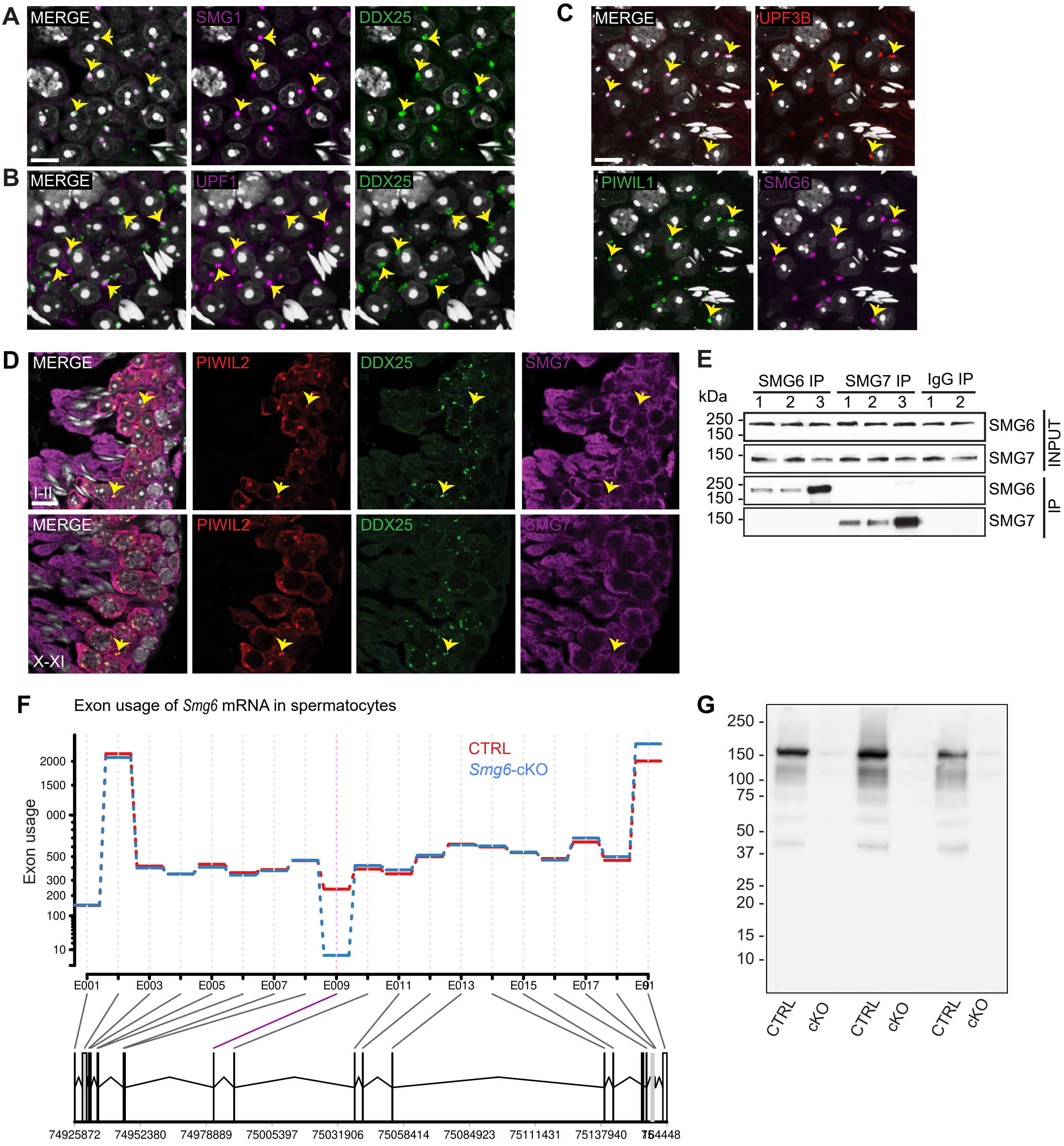

Supplementary Figure S2. Localization of NMD factors during spermatogenesis. (A) SMG1 (magenta) and **(B)** UPF1 (magenta) localize to the CB, visualized by the CB-marker DDX25 (green). Scale bar: 10 µm. **(C)** UPF3B (red) together with SMG6 (magenta) localize to the CB, visualized by the CB-marker PIWIL1 (green). Scale bar: 10 µm. **(D)** SMG7 localization pattern during spermatogenesis at two different stages of the seminiferous epithelial cycle (stages I-II and X-XI). Testis sections were immunostained with SMG7 antibody (magenta) combined with PIWIL2 (red) and DDX25 (green) antibodies. Scale bar: 10 µm. **(E)** Western blot validation of the three replicate anti-SMG6 and anti-SMG7 IP samples (1-3) used in the mass spectrometry analysis. Control IP was done using rabbit IgG. **(F)** Exon usage analysis of *Smg6* gene in CTRL and *Smg6*-cKO mice using RNA-seq data from spermatocytes. The analysis was done by DEXSeq. **(G)** Three control testes and three *Smg6*-cKO testes samples all from different animals were immunoblotted with SMG6 antibody. For all 6 biological samples the whole Western blot membrane is shown.

Supplementary Figure S3

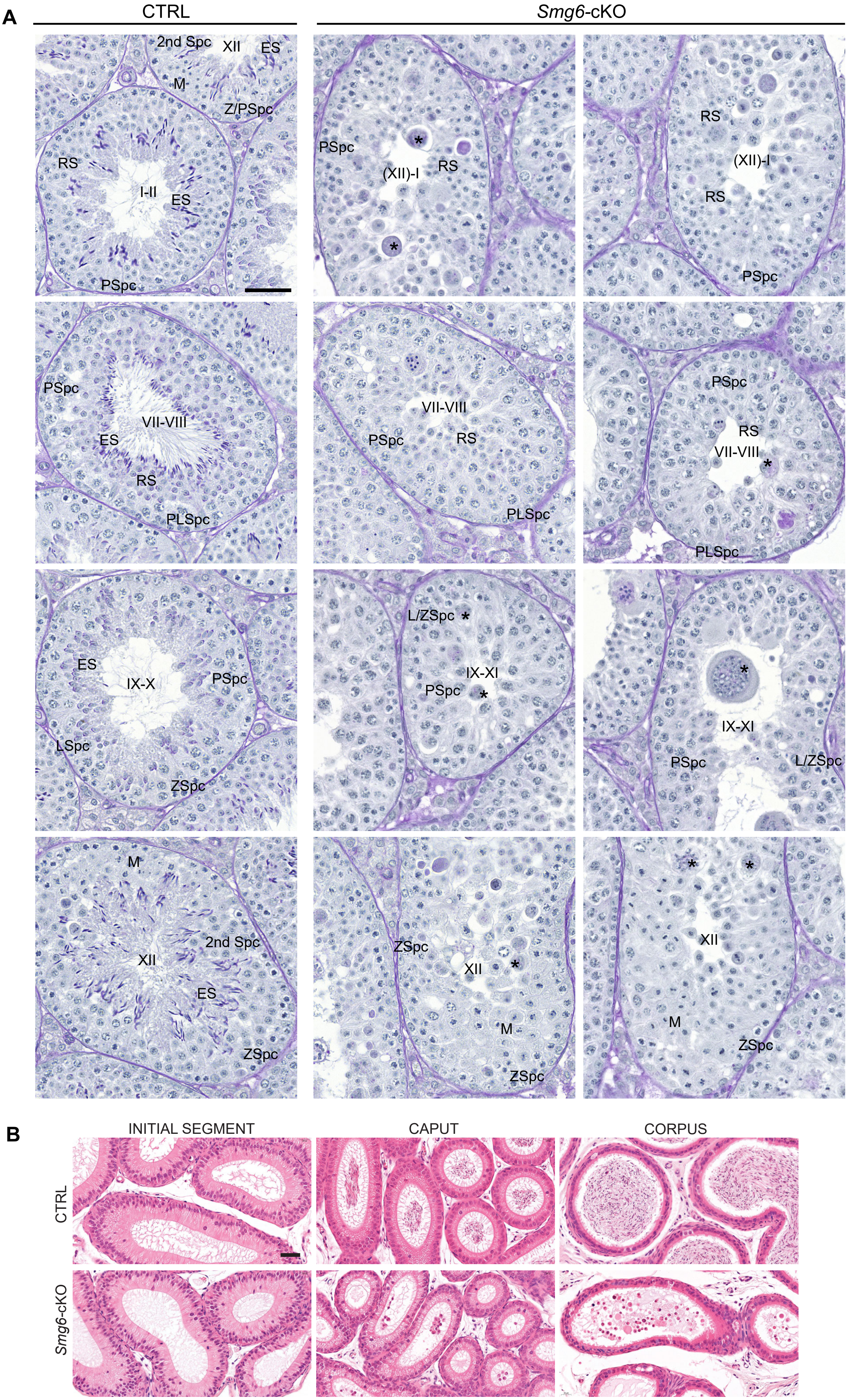

Supplementary Figure S3. Reproductive phenotype of *Smg6*-cKO mice. (A) Bouin-fixed testes sections were stained with PAS staining. Representative images of four different stages of the seminiferous epithelial cycle are shown (stages I-II, VII-VIII, IX-XI and XII). PLSpc, preleptotene spermatocyte; LSpc, leptotene spermatocyte; ZSpc, zygotene spermatocyte; PSpc, pachytene spermatocyte; 2nd Spc, secondary spermatocyte; M, meiotic metaphase plate; RS, round spermatid; ES, elongating spermatid. Asterisks indicate multinucleated round spermatids. Scale bar: 50 μm. **(B)** PFA-fixed paraffin-embedded epididymides of CTRL and *Smg6*-cKO mice were stained with hematoxylin and eosin. Representative images were taken from the initial segment, caput and corpus epididymides. Scale bar: 10 μm.

Supplementary Figure S4

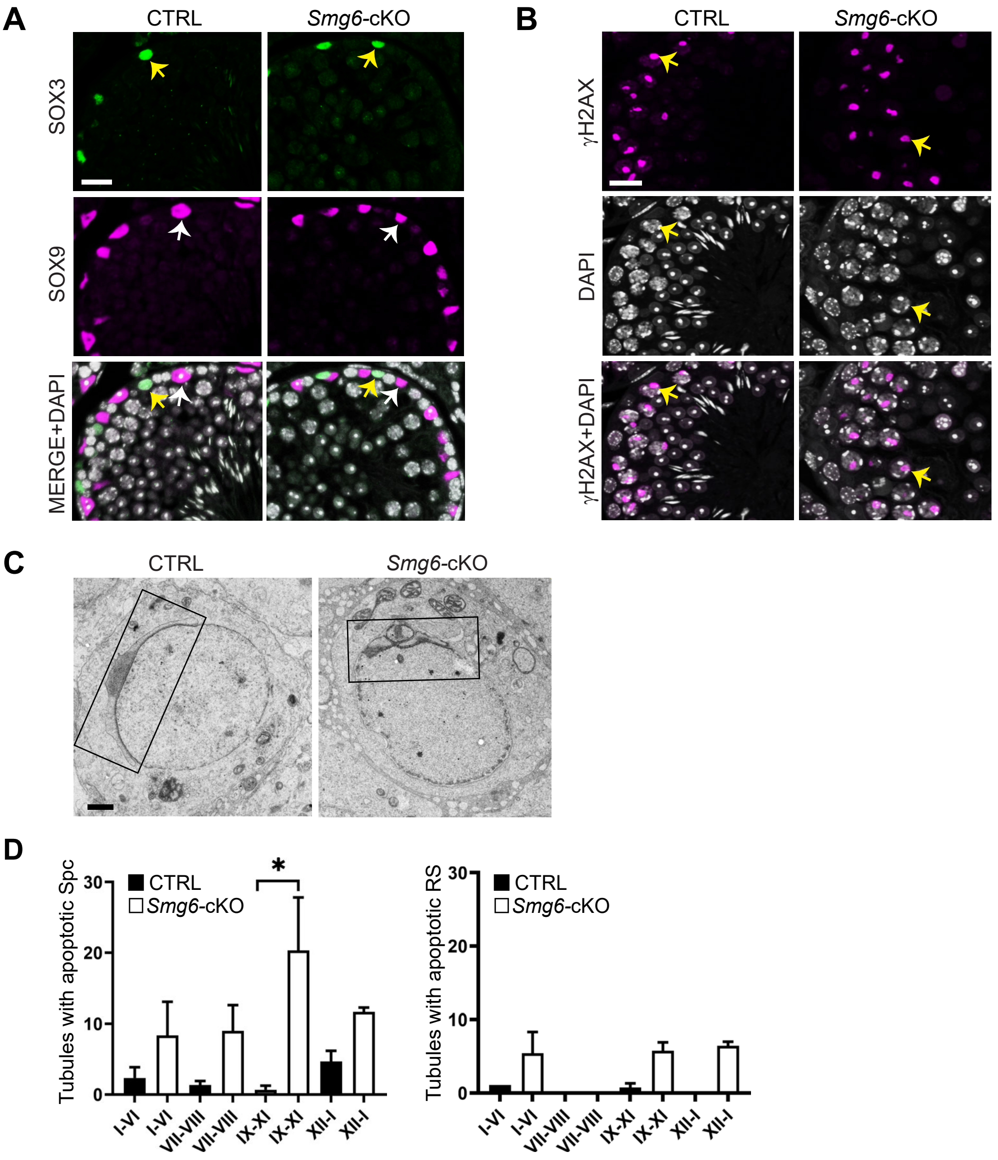

Supplementary Figure S4. Spermatogenic phenotype of *Smg6*-cKO mice. (A) Representative image (stage VII-VIII) of the IF analysis of PFA-fixed paraffin-embedded testis sections with spermatogonia marker anti-SOX3 (green) and Sertoli cell marker anti-SOX9 (magenta). DAPI stains nuclei (white). Yellow and white arrows indicate selected spermatogonia and Sertoli cells, respectively. Scale bar: 20 µm. **(B)** Representative image (stage II-III) of the IF analysis of PFA-fixed paraffin-embedded testis sections with anti-γH2AX (magenta). Yellow arrows indicate selected sex bodies. Scale bar: 20 µm. **(C)** Electron microscopy of round spermatids in control and *Smg6*-cKO testis sections. The acrosomal region inside black squares are shown in higher magnifications in Figure 4B. Scale bar: 1 µm. **(D)** Tubules with apoptotic spermatocytes (left) or round spermatids (right) were counted and categorized based on their stage in the seminiferous epithelial cycle (I-VI, VII-VIII, IX-XI, XII-I). Three biological replicates were analyzed from CTRL and *Smg6*-cKO mice, and Kruskal-Wallis test with Dunn's multiple comparisons was used to determine the significance values between groups. Error bars represent mean±SD of three biological replicates, *P<0.05.

Supplementary Figure S5

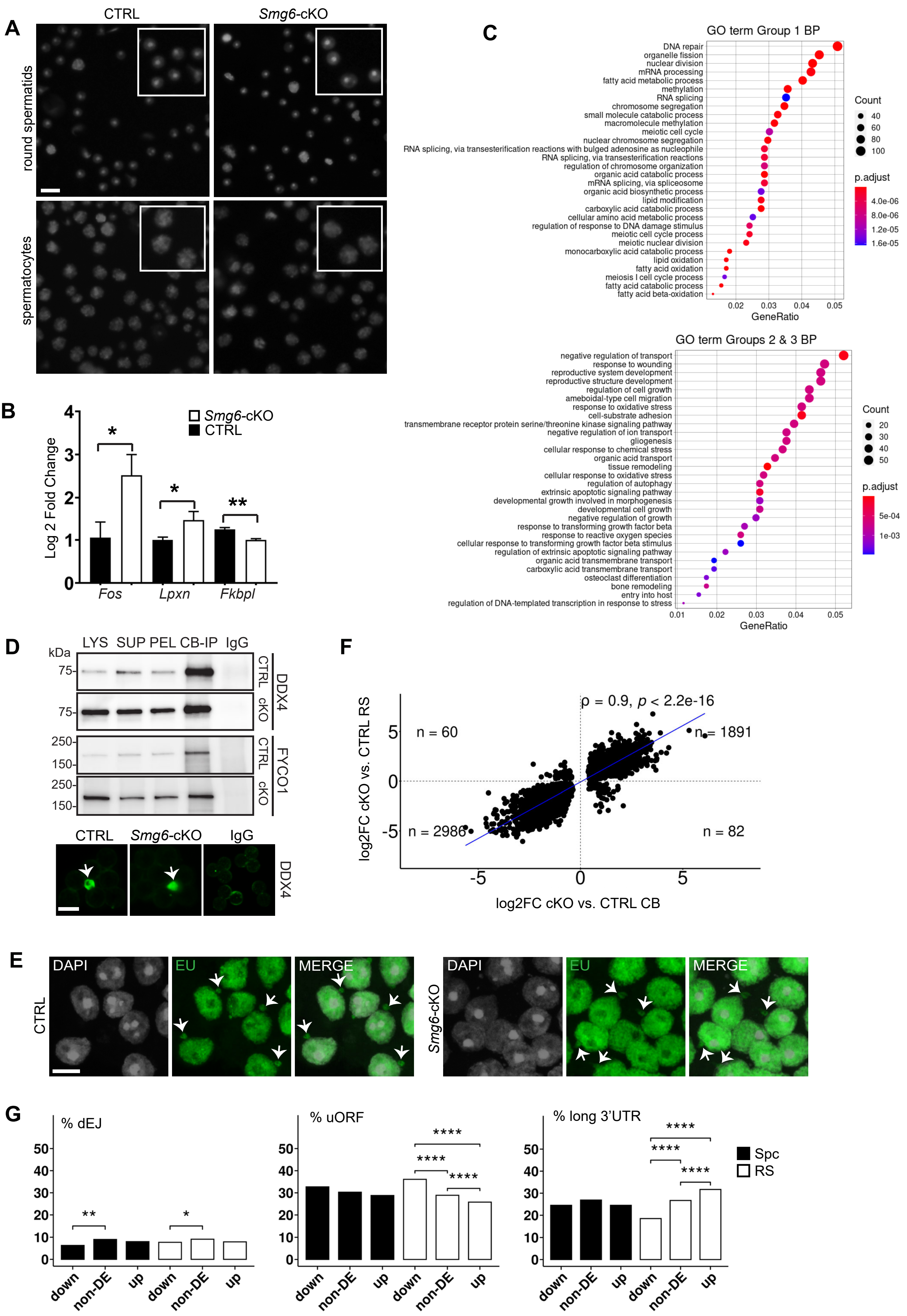

Supplementary Figure S5. Transcriptome analysis of *Smg6*-cKO spermatocytes and round spermatids. (A) Representative DAPI-stained images of pachytene spermatocytes and round spermatid fractions enriched using a BSA-gradient velocity sedimentation method. Scale bar: 20 µm. **(B)** RT-PCR validation of some downregulated (*Fkbpl*, FK506-binding protein-like) or upregulated (*Lpxn*, leupaxin and *Fos*, Proto-oncogene c-Fos) mRNAs in *Smg6*-cKO germ cells. Expression data were normalized to housekeeping genes. Bars represent mean±SD, and p-values are from unpaired t tests. *P<0.05; **P<0.01. Primer sequences are listed in Supplementary Table S7. **(C)** GO term analysis of Group 1 and Group 2+3 genes (see Figure 5F) that have differential expression patterns at the meiotic-to-postmeiotic transition. First 30 Biological Processes were selected for visualization based on adjusted p-values (gradient scale with red indicating the most significant values). GeneRatio: the amount of genes associated with the GO term divided by the total number of genes. Count: the number of genes associated to the GO term illustrated by a dot with a proportional size according to the number of associated genes found in our gene list. Plots were generated in R using clusterProfiler (v3.18.1). **(D)** Western blotting of CBs isolated from CTRL and *Smg6*-cKO testes with anti-DDX4 and anti-FYCO1 antibodies. LYS: cross-linked cell lysate, SUP: supernatant fraction after low-speed centrifugation, PEL: CB-containing pellet fraction after low-speed centrifugation, CB-IP: CB fraction isolated by anti-DDX4 antibody, IgG: negative control IP with rabbit IgG. Panel below: IF with anti-DDX4 antibody (green) to recognize Dynabeads-attached CBs (arrows) isolated from CTRL and *Smg6*-cKO testes. In the negative control IP (IgG), only background staining from beads is detected. Scale bar: 1 µm. **(E)** Stage-specific squash preparations (stage II-V) were incubated with ethynyl uridine (EU) for 10 h, and subsequently, synthesized RNA was visualized using the Click reaction (green). DAPI stains nuclei (white). White arrows indicate selected EU-positive CBs. Scale bar: 10 µm. (**F**) A scatter plot displays the relationship between log2 fold changes (*Smg6*-cKO vs. CTRL) in round spermatid and CB RNA-seq data. The blue line represents the regression line modeling the relationship between data points. The Spearman correlation coefficient is shown as ρ (rho) associated with its significance level (p). **(G)** The percentages of NMD-inducing features (dEJs, uORFs and long 3'UTRs ≥1500 nt) among the downregulated (down, log2FC≤-1.5), the non-differentially expressed (non-DE, log2FC between -1.5 and 1.5), and the upregulated (up, log2FC≥1.5) transcripts in *Smg6*-cKO round spermatids and spermatocytes. Adjusted p-values were generated from a Pearson's Chi-squared test with a fdr post-hoc test.

Supplementary Figure S6

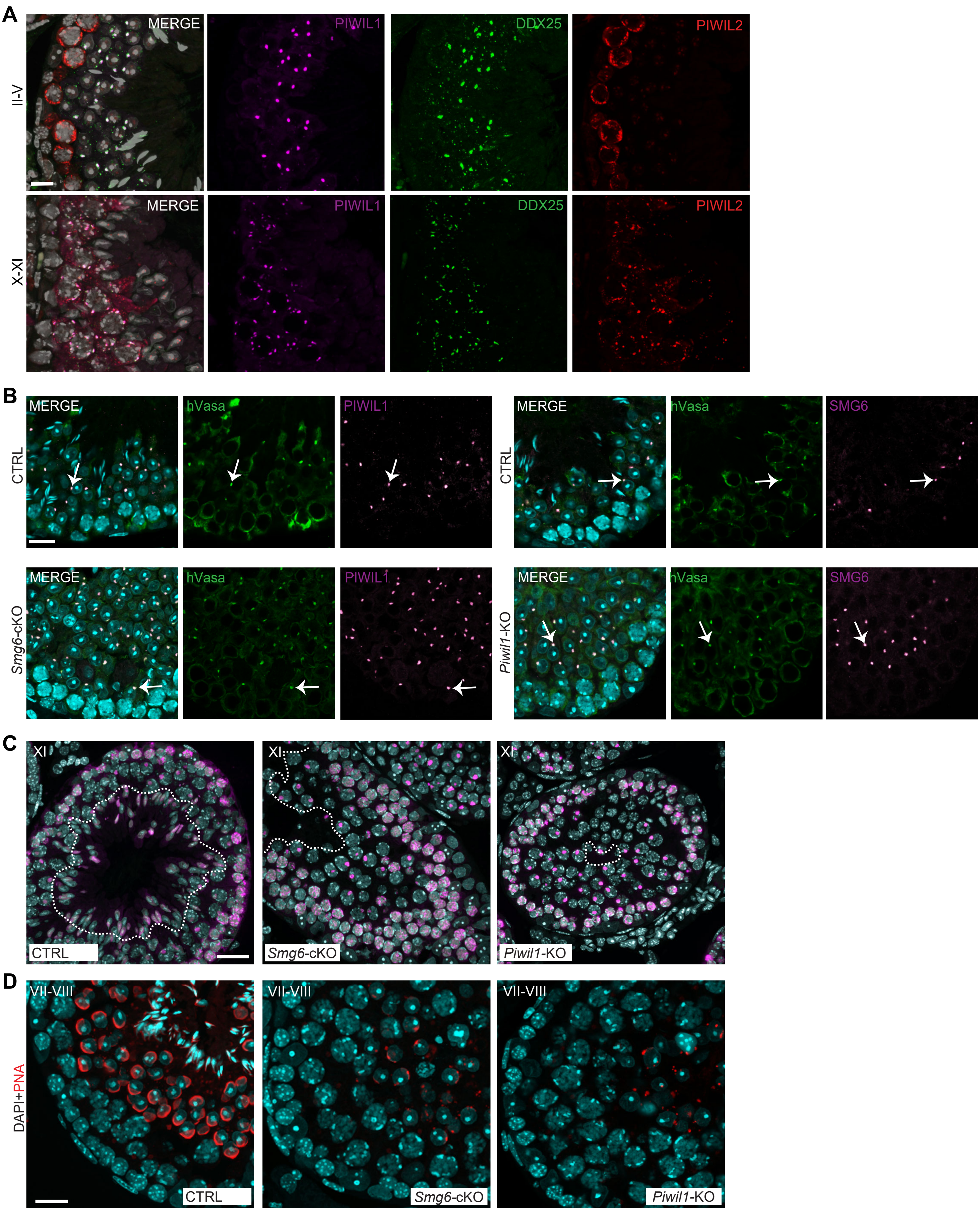

Supplementary Figure S6. Localization and association of SMG6 with PIWIL1. (A) PFA-fixed paraffin-embedded testis sections were immunostained with PIWIL1 (magenta), DDX25 (green) and PIWIL2 (red) antibodies. PIWIL2-positive IMC is prominent in the cytoplasm of early and mid-pachytene spermatocytes (stage II-V). DDX25/PIWIL1/PIWIL2-positive CB precursors appear in stage X-IX pachytene spermatocytes. DDX25 and PIWIL1 are found in the CB in round spermatids at stage II-V. DAPI stains the nuclei (white). Scale bar: 10 µm. **(B)** Localization of PIWIL1/SMG6 (magenta) in CTRL, *Smg6*-cKO and *Piwil1*-KO testis at stage II-V. hVasa (green) was used as a CB marker. Examples of CBs are indicated by white arrows. DAPI stains the nuclei. Scale bar: 10 µm. **(C)** IF of PFA-fixed paraffin embedded testis sections from CTRL, *Smg6*-cKO and *Piwil1*-KO mice was performed to compare their spermatogenic phenotypes at stage XI of the seminiferous epithelial cycle. Antibody against γH2AX (magenta) was used to visualize the layers of zygotene spermatocytes (the whole nucleus stained) and pachytene spermatocytes (nuclear sex body stained). DAPI stains the nuclei. Dashed white line indicates the border between the layers of pachytene spermatocytes and elongating spermatids. Scale bar: 25 µm. **(D)** IF of PFA-fixed paraffin embedded testis sections from CTRL, *Smg6*-cKO and *Piwil1*-KO mice was performed to compare their acrosomal development. PNA (red) was used to visualize the acrosome in round spermatids at stage VII-VIII, DAPI stains the nuclei. Scale bar: 10 µm.

Supplementary Figure S7

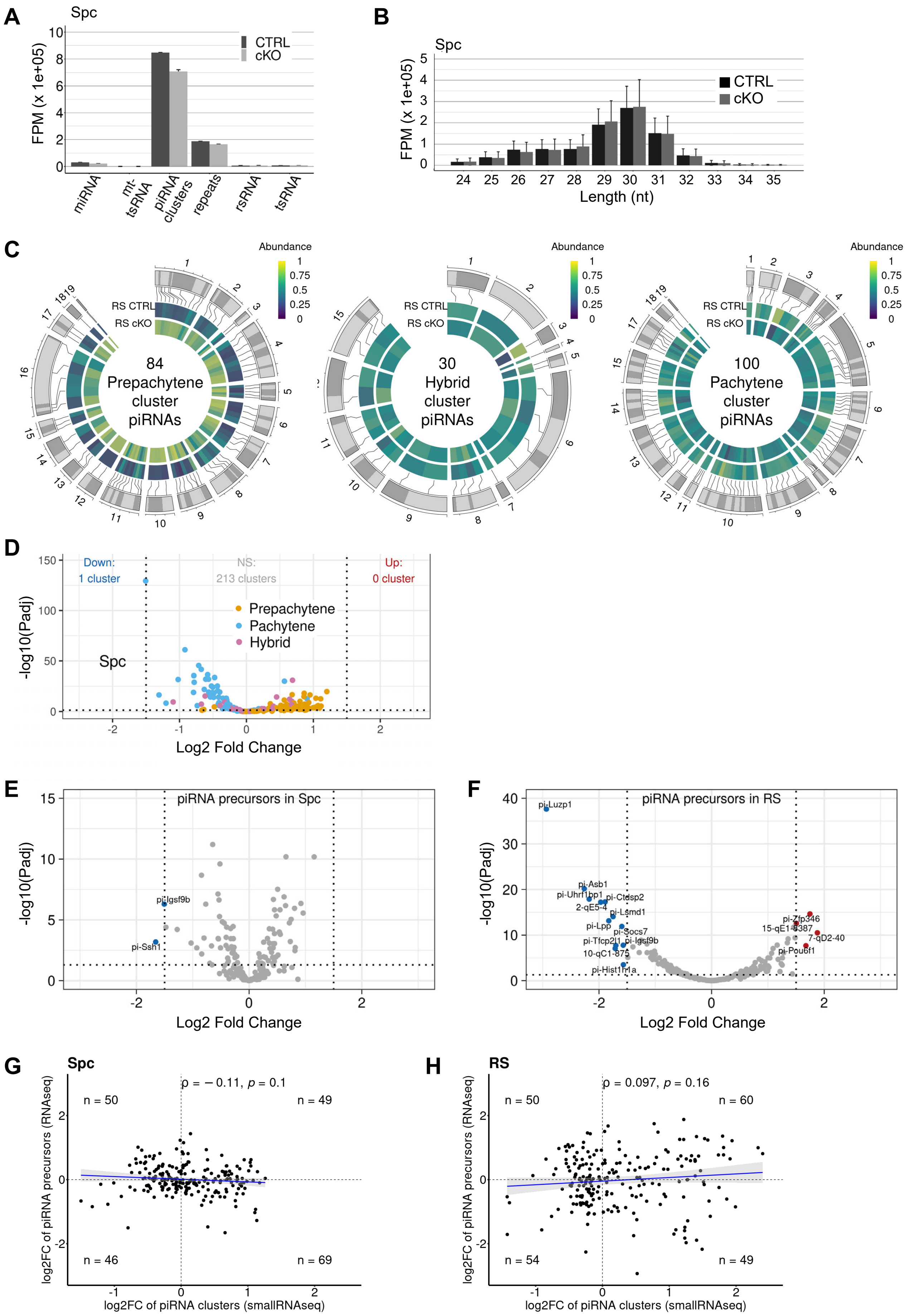

Supplementary Figure S7. Analysis of piRNAs. (A) Distribution of small RNA sequencing reads in genomic regions corresponding to miRNAs, mitochondrial tsRNAs (mt-tsRNA), piRNAs, repeats, ribosomal RNAs (rsRNA) and tsRNAs in CTRL and *Smg6*-cKO (cKO) spermatocytes. Error bars represent mean±SD of three biological replicates. **(B)** Size distribution of piRNA reads in *Smg6*-cKO and control spermatocytes. Raw counts were normalized in FPM. Error bars represent mean±SD of three biological replicates. FPM values in (A) and (B) were calculated using a robust median ratio method using DESeq2 v1.30.0. **(C)** Genome-wide chromosomal distribution (mm10) of small RNA reads within 214 piRNA clusters in control and *Smg6*-cKO round spermatids. Clusters were classified as pre-pachytene (84 clusters), hybrid (30 clusters), and pachytene (100 clusters) according to their abundance from 10.5 to 17.5 dpp (20). Abundance is defined as a group's mean divided by sum of the means (CTRL + cKO). **(D)** Differential expression of pre-pachytene, hybrid and pachytene piRNA clusters (small-RNA-seq) in pachytene spermatocytes. **(E,F)** Volcano plots show differential expression of piRNA precursor transcripts in long-RNA-seq data in spermatocytes (E) and round spermatids (F). **(G,H)** Scatter plots display the relationship between log2 fold changes (*Smg6*-cKO vs. control) of piRNA precursors (analysed from long-RNA-seq data) and piRNA clusters (analysed from small-RNA-seq data) in spermatocytes (G) and round spermatids (H). The blue line represent the regression line modeling the relationship between data points associated with the gray area showing the confidence interval. The Spearman correlation coefficient shown as ρ (rho) associated with its significance level (p).

Supplementary Tables

Supplementary Table S1. (A) Mass spectrometry of SMG6 and SMG7 immunoprecipitations from adult mouse testes. **(B)** Mass spectrometry of CBs isolated from CTRL and *Smg6*-cKO mice.

Supplementary Table S2. Raw and normalized testicular weights from CTRL, HEZ and *Smg6*-cKO mice.

Supplementary Table S3. (A-C) Differential expression analysis of Ensembl-annotated genes in *Smg6*-cKO vs. CTRL spermatocytes (A), round spermatids (B), and CBs (C). Upregulated genes previously identified as NMD targets are indicated. **(D,E)** Stability analysis of statistically significant genes (Padj < 0.05) in *Smg6*-cKO vs. CTRL pachytene spermatocytes (D) and round spermatids (E). **(F,G)** Differential expression analysis of Ensembl-annotated genes in CTRL (F) and *Smg6*-cKO (G) round spermatids vs. spermatocytes.

Supplementary Table S4. (A-C) NMD-inducing features (NIFs) of Ensembl-annotated differentially expressed transcripts in spermatocytes (A), round spermatids (B) and CBs (C). **(D,E)** NIF analysis of meiotic Group 1 transcripts that were downregulated normally in *Smg6*-cKO during the transition from meiotic to postmeiotic stage (from Spc to RS) (D), and Group 2+3 transcripts that resisted the downregulation in *Smg6*-cKO round spermatids (E).

Supplementary Table S5. Differential expression analysis of piRNA clusters from small-RNA-seq data in *Smg6*-cKO vs. CTRL spermatocytes **(A)** and round spermatids **(B)**.

Supplementary Table S6. Differential expression analysis of piRNA cluster transcripts in *Smg6*-cKO vs. CTRL spermatocytes (**A**), round spermatids (**B**) and CBs (**C**). TEtranscripts analysis of genes and transposable elements in *Smg6*-cKO vs. CTRL spermatocytes (**D**), round spermatids (**E**) and CBs (**F**).

Supplementary Table S7. Primers for genotyping and RT-qPCR.
